# Resistance training alleviates muscle atrophy and muscle dysfunction by reducing inflammation and regulating compromised autophagy in aged skeletal muscle

**DOI:** 10.3389/fimmu.2025.1597222

**Published:** 2025-06-03

**Authors:** Yangfan Cao, Jiawei Zhou, Helong Quan, Wei Li, Ting Li, Lifeng Wang

**Affiliations:** ^1^ College of Physical Education and Health Sciences, Zhejiang Normal University, Jinhua, China; ^2^ College of Physical Education, Northeast Normal University, Changchun, China

**Keywords:** aging, skeletal muscle, mTORC1, chronic inflammation, autophagy

## Abstract

**Background:**

Age related muscle atrophy is associated with chronic inflammation and impaired autophagy. Resistance training serves as an effective intervention for enhancing skeletal muscle hypertrophy.

**Methods:**

This study utilized a naturally aged mouse model to investigate the role of the mammalian target of rapamycin complex 1 (mTORC1) pathway in mediating the effects of resistance training on chronic inflammation and autophagy in aged skeletal muscle.

**Results:**

Our findings demonstrate that resistance training increased the wet weight of the gastrocnemius (GAS) and quadriceps (Quad), absolute number of fibers and the cross-sectional areas (CSA) of skeletal muscles, as well as enhanced the maximum load and maximum grip strength. These findings indicate that resistance training improved the quality and strength of skeletal muscles in aging mice. Resistance training alleviated inflammation in aged skeletal muscle by promoting M2 macrophage polarization, reducing the mRNA levels of tumor necrosis factor alpha (TNF-α), nuclear factor-kappaB (NF-κB) and interleukin-1beta (IL-1β), and increasing the mRNA levels of interleukin-6 (IL-6) and interleukin-10 (IL-10). In aged skeletal muscle, resistance training decreased the protein expression of mTOR, regulatory-associated protein of mTOR (Raptor), p70 ribosomal protein s6 kinase (p70S6K), IL-1β, and hypoxia-inducible factor 1-alpha (HIF-1α) without affecting protein kinase B (AKT) activity. Moreover, autophagy, which is reduced in aged muscle, was increased by resistance training through increased AMP-activated protein kinase (AMPK) activity and increased BCL-2-interacting protein 1 (Beclin1) and transcriptional factor EB (TFEB) expression.

**Discussion:**

Our study suggests that resistance training was associated with alleviated inflammation and regulated autophagy, potentially involving the mTORC1-HIF-1α and mTORC1-AMPK pathways, which may contribute to improved skeletal muscle mass in aged mice.

## 1 Introduction

Aging induces a complex degenerative process in skeletal muscles, resulting in diminished muscle mass, strength, and function, potentially leading to sarcopenia (1, 2). Impaired macroautophagy and chronic inflammation are hallmarks of aging and are accompanied by a reduction in muscle mass (3–5). In the elderly, persistently elevated levels of pro-inflammatory cytokines, including tumor necrosis factor alpha (TNF-α) and interleukin-1 beta (IL-1β), promote proteolysis and dysregulation of tissues and organ systems, contributing to skeletal muscle atrophy (6–9). During the aging process, dysregulated autophagy leads to a significant accumulation of damaged cellular components (such as defective mitochondria), resulting in a dysregulated internal environment of the skeletal muscle (10). Excessive activation of the mammalian target of rapamycin complex 1 (mTORC1) promotes inflammation and impairs autophagy (11, 12). Resistance training combats sarcopenia by enhancing muscle protein synthesis and suppressing proteolysis, demonstrating superior efficacy in muscle hypertrophy compared to other trainings (13, 14). Recent investigation has demonstrated that resistance training could reduce the activation of the mTORC1 pathway and mitigate age-related muscle loss (15, 16). Moreover, resistance exercise has demonstrated beneficial effects in mitigating age-related chronic inflammation and stimulating autophagy (17, 18).

mTORC1 regulates several cellular physiological processes and its dysregulation during aging affects inflammation and autophagy (19). Excessive activation of mTORC1 promotes macrophage polarization toward the M1 phenotype, thereby exacerbating inflammation (20, 21). Additionally, it stimulates IL-1β production through the activation of glycolysis, and IL-1β subsequently enhances hypoxia-inducible factor 1-alpha (HIF-1α) expression (22, 23). Studies have shown that HIF-1α upregulated the expression of pro-inflammatory mediators by binding to the IL-1β promoter and activating nuclear factor kappa B (NF-κB) in macrophages (24–26). Overactivation of mTORC1 reduces autophagy (27, 28). It has been demonstrated that constant activation of mTORC1 reduces autophagy by inhibiting Unc-51 like autophagy activating kinase 1 (ULK1), and treatment of tuberous sclerosis complex 1 (TSC1) muscle-specific knockout (TSCmKO) mice with rapamycin is sufficient to restore autophagy (27). However, the role of mTORC1 in regulating both autophagy and inflammation in skeletal muscle during aging remains unclear.

In this study, we used a naturally aging mouse model to investigate the effect of the mTORC1 pathway in mediating the effects of resistance exercise on chronic inflammation and autophagy in skeletal muscle during aging. Our results demonstrated that resistance training alleviates chronic inflammation and enhances autophagy in aged skeletal muscle by downregulating overactive mTORC1. The underlying mechanisms are associated with the mTORC1-HIF-1α and mTORC1-autophagy pathways. These findings provide experimental evidence for the role of resistance training in mitigating age-related skeletal muscle loss, and offer light on potential therapeutic strategies for age-related muscle atrophy.

## 2 Materials and methods

### 2.1 Animals

Six-week-old male C57BL/6J mice (n=40) were purchase from Gempharmatech (Nanjing, China). The mice were maintained in a climate-controlled facility (temperature 20-25°C, humidity 50-60%, 12-h photoperiod) with ad libitum access to food and water. The mice were fed an SPF-grade standard maintenance rodent diet provided by Xietong Bio-Engineering (#1010088). Each cage housed five mice. Follow the acclimation period, animals were randomized into four groups (n=10 per group): Young Control (YC), Young Resistance Training (YR), Old Control (OC), and Old Resistance Training (OR). The research was conducted in accordance with ethical guidelines and provide relevant details of the ethical approval obtained. All animal experiments were approved by the Animal Management Committee of Zhejiang Normal University (ZSDW2022028, 2022). The YC and YR groups commenced interventions at 8 weeks of age, whereas the OC and OR groups began at 18 months of age. Functional tests were conducted before the first training session and after the final session. Subsequently, following a 24-h fasting period, the animal was anesthetized with a 25% urethane solution, euthanized. The soleus (SOL), gastrocnemius (GAS), tibialis anterior (TA), and quadriceps (Quad) were weighed using an analytical balance. Muscle samples were immediately dissected, snap-frozen in liquid nitrogen, and stored at -80°C until analysis.

### 2.2 Training protocol

As an effective resistance training intervention method, ladder climbing can effectively promote skeletal muscle hypertrophy (29, 30).In this study, the YR and OR groups underwent an 8-week training protocol. The training apparatus consisted of a ladder (110×18 cm, 85° incline, 1.5 cm rung spacing) topped with a dark chamber (20×20×20 cm). Prior to training, the mice completed a 7-day acclimation period, during which weights were attached to their tails, and climbing was encouraged successful climb was followed by a 120-s rest in the dark chamber. This process was repeated until the mice voluntarily climbed the ladder three consecutive times without stimulation. The formal intervention comprised 24 training sessions over 8 weeks (Mondays, Wednesdays, and Fridays). Each training session included four adaptive climbs at 50%, 75%, 90%, and 100% of the maximum load, followed by five sets of formal climbs, each comprising four repetitions. The load for formal climbs was incrementally increased by 5 g set, with a 2-min inter-set and 60-s inter-repetition rest period (31).

### 2.3 Muscle function

Resistance training–induced skeletal muscle hypertrophy in young mice has been consistently demonstrated in numerous studies; therefore, the present study focused specifically on aging skeletal muscle to investigate age-related differences in the adaptive response to resistance training (32, 33).

The maximum load capability of the mice was assessed one day prior to the formal intervention and one day after completion. The climbing height was set at 30 cm, with an initial load of 50% of the body weight. After each successful climb, mice were allowed to rest for 120 s in the dark chamber. Subsequently, the load was incrementally increased by 10% of the body weight until task failure. Failure to complete three consecutive trials was defined as a test failure, and the successfully lifted maximal load was recorded as the maximum load capability. The resistance training protocol was optimized based on previous research findings and tailored to meet the needs of this study (31).

The maximum grip strength was evaluated one day before the formal intervention and one day after completion. Mice underwent three acclimation trials with a 5-gram weight attached (excluding the weight of the sponge net) to familiarize them with the procedure. The experimental apparatus consisted of a sponge net and steel balls of various weights. Once the weight was secured, the experimenter lifted the mouse by its tail and recorded the time the mouse maintained its grip on the sponge net with its limbs (34). A grip lasting longer than 3 s was recorded as a success. Failure to lift the net in three attempts was considered a test failure, and the weight of the last successful lift was noted. The initial weight for the formal test was 20 g, which was increased by 10 g after each trial. Upon failure, the median weight from the last two trials was used for subsequent tests until successful completion.

### 2.4 Hematoxylin and eosin staining

Following mice dissection, gastrocnemius (GAS) samples were harvested, embedded, immediately frozen in liquid nitrogen, and stored at -80°C before sectioning into 12 μm sections. The tissue sections were stained with hematoxylin-and eosin (H & E) (Solarbio Science & Technology, Beijing, China) following the manufacturer’s instructions. The skeletal muscle morphology of each group was observed at ×200 and ×400 magnification using a German Leica fluorescence microscope. The CSA of individual muscle fibers was quantified using ImageJ software (Maryland, USA). For each sample, 30 randomly selected muscle fibers were manually outlined and measured. All measurements were performed by an investigator blinded to the group allocation to minimize bias. Each sample was measured at ×400 magnification and the average value was normalized to the reference standard (error margin ±1.5%).

Skeletal muscle sections were processed as previously described, and sections were stained with H&E staining. Fiber count was performed using ImageJ software (Maryland, USA). For each muscle sample, at least three cross-sectional images were taken from different regions of the muscle to ensure representative counting. Muscle fiber number was determined by averaging the counts across these images. For statistical analysis, fiber number was expressed as the total number of fibers per area to account for differences in section thickness or region of sampling. Statistical differences between groups were analyzed using two-way ANOVA followed by Tukey’s *post-hoc* test.

### 2.5 qRT-PCR

Total RNA was extracted from the quadriceps (Quad) muscle using TRIzol reagent (Thermo Fisher, 15596026). RNA was reverse-transcribed into cDNA using the High-Capacity cDNA Reverse Transcription Kit (Takara, DRR037A). Fast SYBR Green Master Mix (Takara, DRR037A) was used for the real-time PCR quantification of gene expression. Primer sequences are listed in **Table 1**. GAPDH served as an internal control. The relative expression data were calculated according to the 2^−ΔΔCt^ method and presented as relative expression levels (35).

**Table 1:** Sequences of forward and reverse primers used for qRT-PCR.

Gene	Primer forward	Primer reverse
GAPDH	ACCCTTAAGAGGGATGCTGC	CCCAATACGGCCAAATCCGT
TNFα	GATCGGTCCCCAAAGGGATG	CCACTTGGTGGTTTGTGAGTG
NK-κB	CTCTGGCACAGAAGTTGGGT	TCCCGGAGTTCATCTCATAGT
IL-1β	TGCCACCTTTTGACAGTGATG	TGATGTGCTGCTGCGAGATT
IL-6	GACAAAGCCAGAGTCCTTCAGA	TGTGACTCCAGCTTATCTCTTGG
IL-10	GGTAGAAGTGATGCCCCAGG	ACACCTTGGTCTTGGAGCTTAT

### 2.6 Immunofluorescence staining

CD206 is a specific marker of M2 macrophages, mediating the uptake of extracellular substances for clearance or antigen presentation (36, 37). iNOS defines M1 macrophages through its nitric oxide–mediated antimicrobial activity and is widely regarded as a standard marker of classical activation (38).

The quadriceps served as an ideal tissue for studying muscle atrophy mechanisms due to its rapid age-related degeneration that directly impairs mobility (39). Tissue sections were fixed in 4% paraformaldehyde for 10 min, rinsed with PBS, and permeabilized with 0.5% Triton X-100 (20 min). Subsequently, the sections were blocked in 1% BSA solution diluted in PBST (PBS + 22.52 mg/mL Glycine + 0.1% Tween 20) for 30 min at room temperature. Then, tissue sections were performed overnight at 4°C with the following primary antibodies (1% BSA + PBST): iNOS (Proteintech, 18985-1-AP) and CD206 (Proteintech, 18704-1-AP). After three 5-min PBS washes, sections were incubated with species-matched secondary antibodies: goat anti-mouse IgG conjugated with Alexa Fluor 488 (Beyotime Biotechnology, #A0428) and goat anti-rabbit IgG conjugated with Alexa Fluor 594 (Abcam, #ab150080) in blocking buffer for 60 minutes under light-protected conditions. Following three PBS washes, the sections were incubated for 1 h in the dark at room temperature with Alexa Fluor 488-conjugated goat anti-mouse (Beyotime, #A0428) and Alexa Fluor 594-conjugated goat anti-rabbit secondary antibodies (Abcam, #ab150080). Cell nuclei were counterstained with 4’, 6-diamidino-2-phenylindole (DAPI) (Beyotime Institute of Biotechnology), and fluorescence imaging was conducted using a Leica fluorescence microscope.

### 2.7 Western blot

Quad tissues were mechanically homogenized in ice-cold RIPA lysis buffer (Thermo Scientific, A32959) containing protease and phosphatase inhibitor mixture. Following centrifugation at 13, 000 rpm for 15 min at 4°C, supernatants were collected and protein concentrations were determined using a BCA assay kit (Thermo Scientific, 23225). Protein samples (30 μg/lane) were separated by sodium dodecyl sulfate-polyacrylamide-polyacrylamide gel electrophoresis (SDS-PAGE) and transferred to polyvinylidene fluoride (PVDF) membranes. Membranes were blocked at room temperature for 1 h with 5% non-fat dry milk (Bio-Rad, 1706404) in Tris-buffered saline with Tween-20 (TBST) and incubated overnight at 4°C with primary antibodies: p-AKT (Ser473) (Cell Signaling Technology, #86758, 1:1000), AKT (Cell Signaling Technology, #9272, 1:1000), p-AMPKα (Thr172) (Cell Signaling Technology, #2535, 1:1000), AMPKα (Cell Signaling Technology, #2532, 1:1000), mTOR (Cell Signaling Technology, #2983, 1:1000), Raptor (Cell Signaling Technology, #2280, 1:1000), p70S6K (Cell Signaling Technology, #9202, 1:1000), IL-1β (Proteintech, 16806-1-AP, 1:2000), HIF-1α (Cell Signaling Technology, #14179, 1:1000), Beclin1 (Proteintech, 11306-1-AP, 1:1000), TFEB (Proteintech, 13372-1-AP, 1:1000), p62 (Proteintech, 18420-1-AP, 1:1000), LC3 (Cell Signaling Technology, #2775, 1:2000), and GAPDH (Proteintech, 60004-1-Ig). All experiments used the same protein marker (Bio-Rad, #1610374). Following three TBST washes, the membranes were incubated with secondary antibodies (goat anti-mouse for Proteintech Cat RGAM001, 1:5000; goat anti-rabbit for Cell Signaling Technology Cat #7074, 1:2000) for 2 h at room temperature. After three subsequent washes with TBST, protein bands were detected using an enhanced chemiluminescence solution (Thermo Scientific, 34578), and the membrane was imaged using a chemiluminescence imaging system (Bio-Rad). Protein expression levels were quantified using the ImageJ software. The numerical annotations on the blot margins indicate the actual molecular weights of the prestained protein ladder markers visible on the gel. All representative imprint image reflects the trends of multiple independent experiments. The selection criterion for the representative image is the typical result that is consistent with the trend of the population data.

### 2.8 Statistical analysis

Statistical analyses were performed using IBM SPSS Statistics 19 (Chicago, USA), with graphical representations generated in Origin 2021 (Hampton, USA). The experimental results were expressed as mean ± SEM. A paired t-test was used for comparisons between groups. Differences among multiple groups were assessed using two-way analysis of variance (ANOVA), followed by *post-hoc* comparisons with Tukey’s test. Statistical significance was set at p < 0.05.

## 3 Results

### 3.1 Resistance training increased wet weight and function and ameliorated CSA loss in aged skeletal muscle

**Figure 1:**
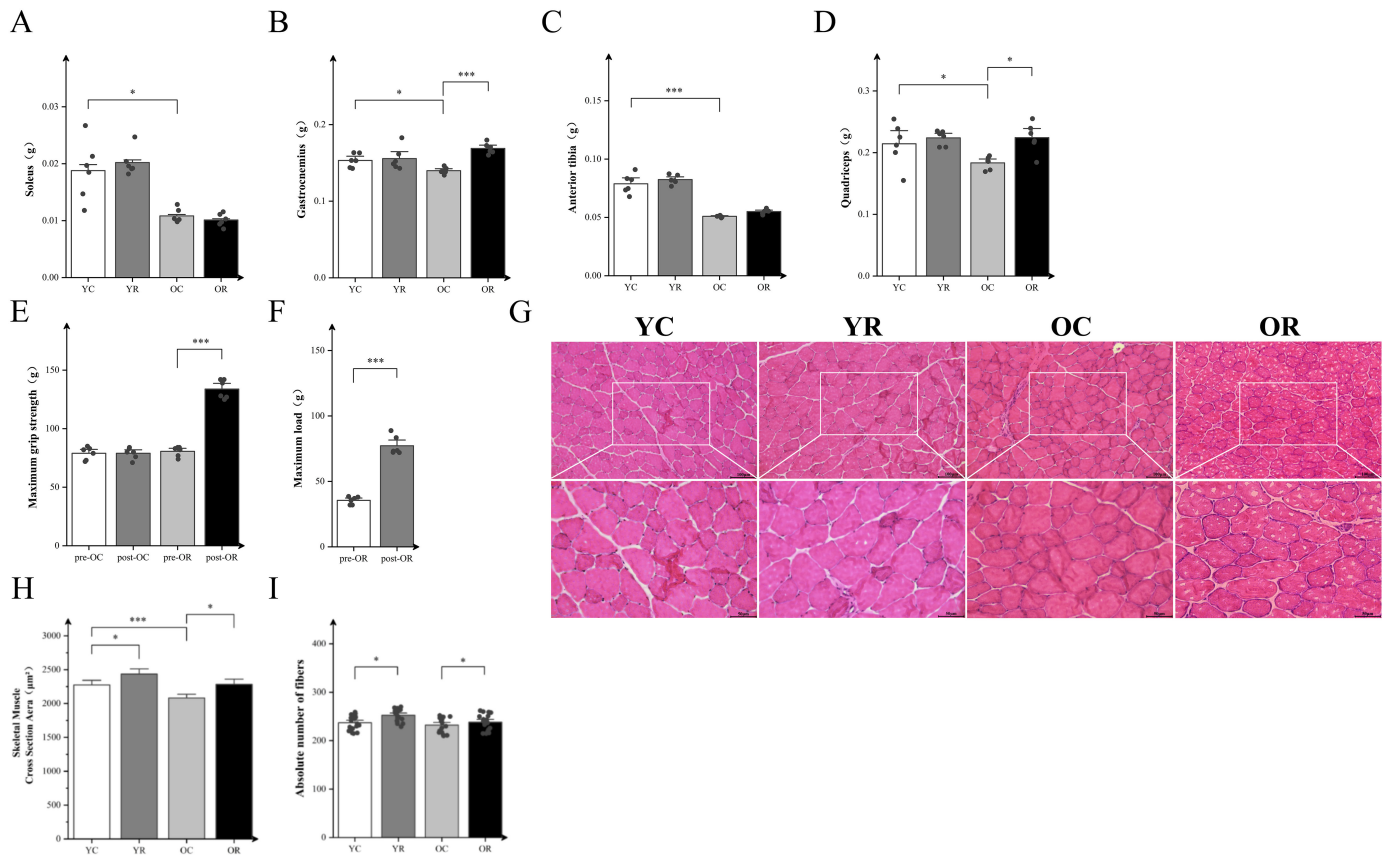
Resistance Training increased wet weight, function and ameliorated CSA loss in aged skeletal muscle. **(A–D)** Wet weights of the soleus (SOL), gastrocnemius (GAS), tibialis anterior (TA), and quadriceps (Quad). **(E)** Maximum grip strength, “pre” and “post” refer to before and after training. **(F)** Maximum load, “pre” and “post” refer to before and after training. **(G)** H&E staining of GAS. **(H)** Cross-sectional area (CSA) of muscle fibers (μm²). **(I)** The absolute number of fibers in GAS. Pre- and post-resistance training in mice were analyzed by paired t-test. The remaining data were analyzed by Two-way ANOVA with Tukey *post hoc* test. Scale bars, 100 µm and 50 µm. All data are presented as means ± SEM, n=6 per group. *p < 0.05, ***p < 0.001.

We reported the body weights of mice in each group and the wet weights of soleus (SOL), gastrocnemius (GAS), tibialis anterior (TA), and quadriceps (Quad) in **Table 2**. The present investigation employed an aged mice model to evaluate the impact of resistance training intervention on senescent skeletal muscle. Resistance training had no significant effect on skeletal muscle wet weight in the YR group compared to YC controls, as observed in the SOL, GAS, TA, and Quad (p > 0.05; **Figures 1A–D**). In contrast, the OC group exhibited significantly reduced weights of SOL, GAS, TA, and Quad relative to the YC group (p < 0.05). Notably, resistance training enhanced the weight of both GAS and Quad (p < 0.05). Additionally, the maximum load and grip strength were enhanced in aged mice after training (**Figures 1E, F**). Meanwhile, we used HE staining to further observe the muscle cross section area (CSA) and the number of myofibers (**Figures 1G–I**). Resistance training significantly increased the CSA of skeletal muscle in YR group compared to the YC group (p < 0.05). The CSA were significantly decreased during aging (p < 0.001) and increased by resistance training (p < 0.05). **Figure 1I** showed that aging did not significantly affect the absolute number of muscle fibers (p > 0.05), whereas resistance training significantly increased it in both young and old mice (p < 0.05). The above results revealed that skeletal muscle atrophy occurs during aging, and resistance training could alleviate it.

**Table 2:** The body weights and wet weights of mice in each group.

Groups	Body Weight (g)	Soleus (g)	Gastrocnemius (g)	Tibialis anterior (g)	Quadriceps (g)
YC	30.67 ± 1.37	0.0188 ± 0.0023	0.1532 ± 0.0032	0.0788 ± 0.0037	0.2093 ± 0.0136
YR	28.83 ± 1.17	0.0202 ± 0.0009	0.1556 ± 0.0057	0.0824 ± 0.0016	0.2204 ± 0.0043
OC	34.67 ± 1.64	0.0108 ± 0.0005	0.1398 ± 0.0017	0.0509 ± 0.0005	0.1932 ± 00059
OR	32.33 ± 1.51	0.0101 ± 0.0004	0.1689 ± 0.0032	0.0543 ± 0.0008	0.2243 ± 0.0098

The body weights of mice in each group and the wet weights of soleus (SOL), gastrocnemius (GAS), tibialis anterior (TA), and quadriceps (Quad). All data are presented as means ± SEM, n=6 per group.

### 3.2 Resistance training promoted macrophage polarization toward the anti-inflammatory phenotype in aged skeletal muscle

**Figure 2:**
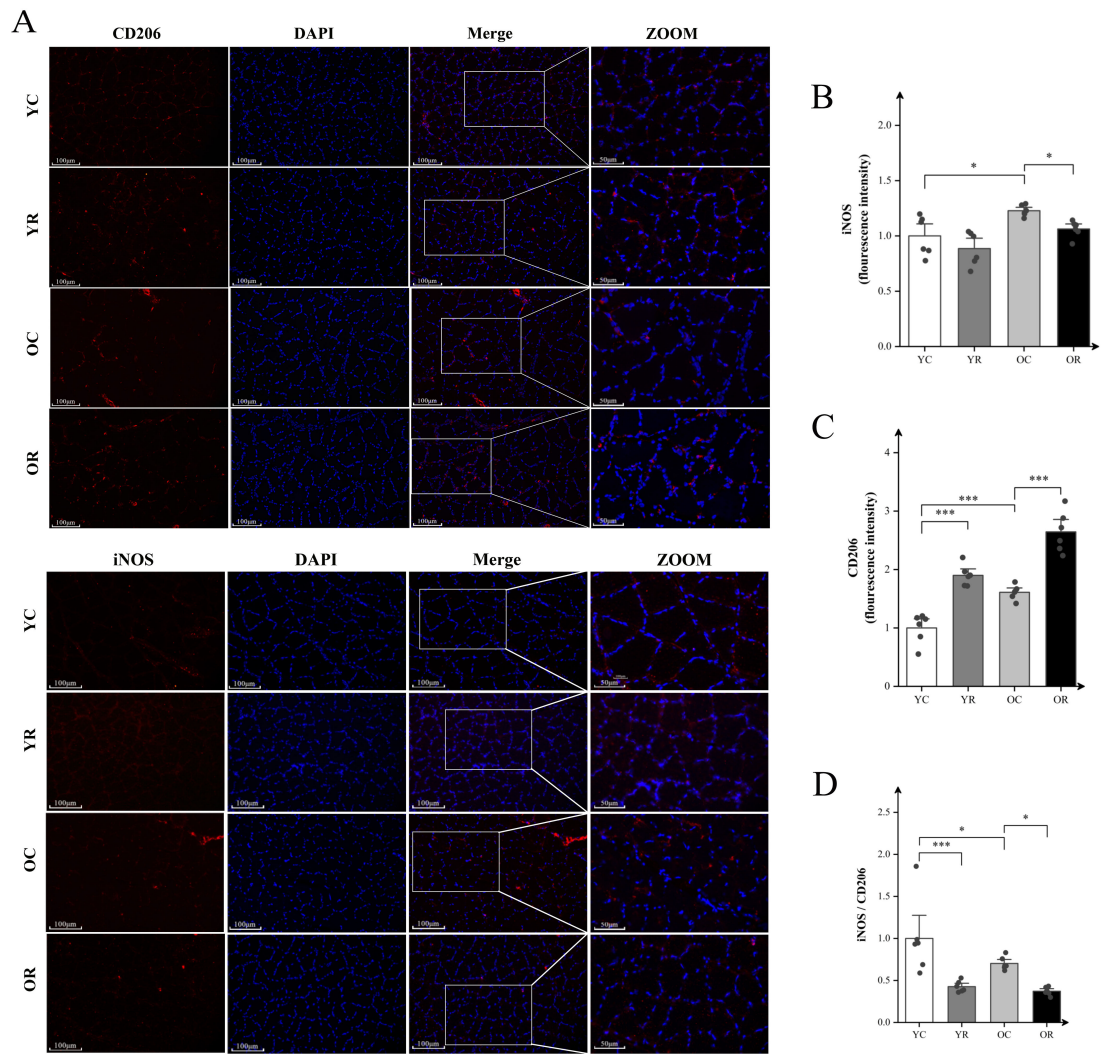
Resistance training promoted polarization macrophages toward the anti-inflammatory phenotype in aged skeletal muscle. **(A)** Representative fluorescence images of CD206 and iNOS in Quad. **(B)** Fluorescence intensity expression of iNOS. **(C)** Representative fluorescence images of CD206. **(D)** The ratio of iNOS/CD206. Scale bars, 100 µm and 50 µm. Four random regions were quantified for each sample. All data are presented as means ± SEM (Two-way ANOVA with Tukey *post hoc* test), n=6 per group. *p < 0.05, ***p < 0.001.

To assess the inflammatory microenvironment in aged skeletal muscle, immunofluorescence analysis was conducted using macrophage markers: iNOS for M1 polarization and CD206 for M2 polarization status (**Figure 2A**). Fluorescence intensity analysis revealed that in comparison to the YC group, the YR group exhibited no significant difference in fluorescence intensity of iNOS (p > 0.05), whereas the CD206 was markedly elevated (p < 0.001; **Figures 2B–D**). The iNOS (p < 0.05) and CD206 (p < 0.01) in the OC group were significantly higher than YC group, while the ratio of iNOS/CD206 was decreased (p < 0.05). In aged muscle, resistance training markedly upregulated CD206 expression (p < 0.001), concomitant with reduced iNOS expression (p < 0.05) and lowered iNOS/CD206 ratio (p < 0.05). The above observation results regarding iNOS and C206 can reflect that resistance training further promotes the polarization of macrophages towards the M2 anti-inflammatory phenotype while inhibiting M1 phenotype polarization in aged skeletal muscle.

### 3.3 Resistance training attenuated mRNA levels of inflammatory factors in aging skeletal muscles

**Figure 3:**
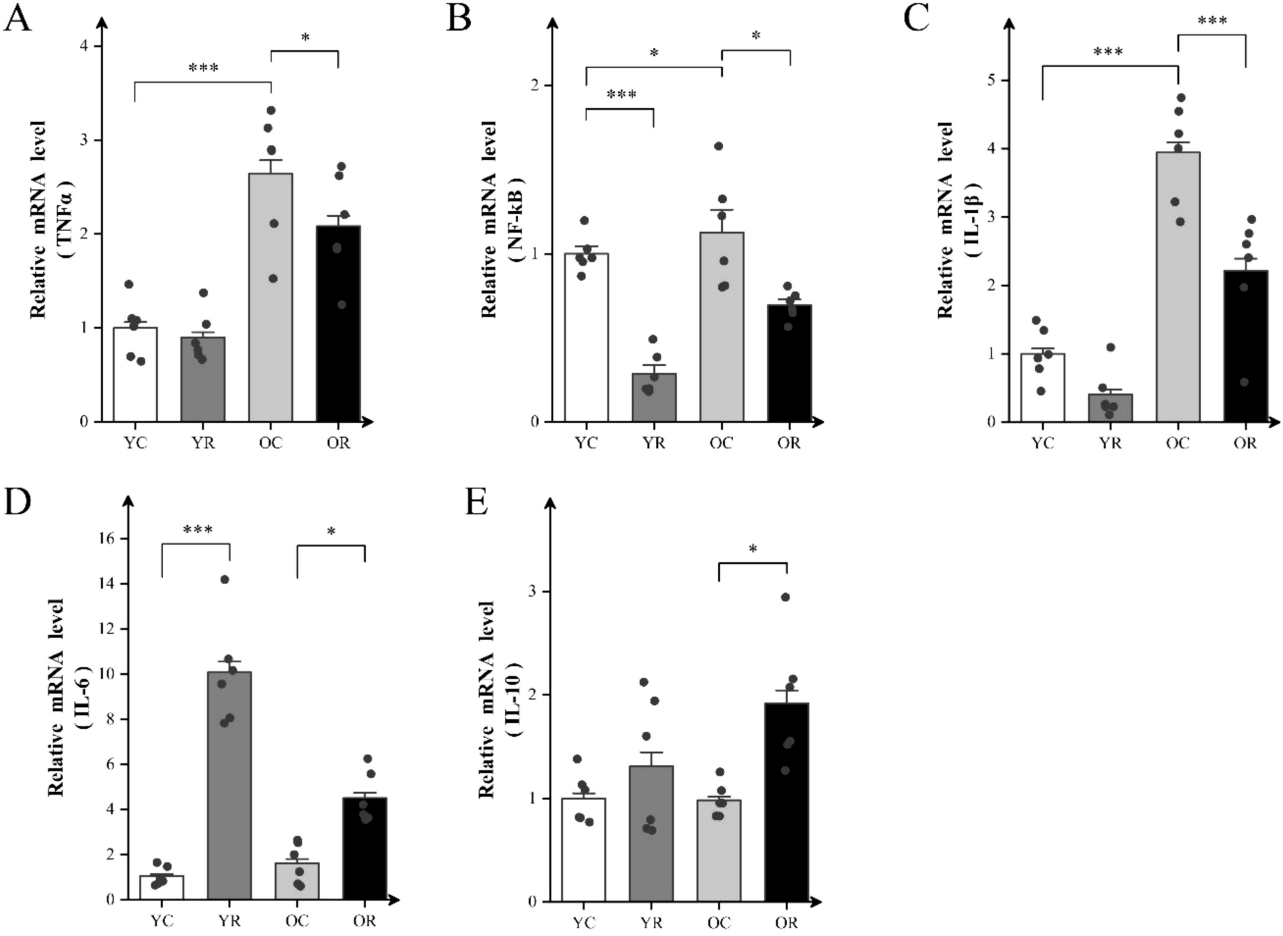
Resistance training attenuated mRNA levels of inflammatory factors in aging skeletal muscle. **(A–E)** The mRNA expression of TNFα, NF-kB, IL-1β, IL-6 and IL-10 in Quad. All data are presented as means ± SEM (two-way ANOVA with Tukey *post hoc* test), n=6 per group. *p < 0.05, ***p < 0.001.

We further examined the effects of resistance training on inflammatory factors levels using qRT-PCR. Resistance training significantly elevated the mRNA levels of NF-κB and IL-6 in the YR group compared with the YC group (p < 0.001; **Figures 3A–C**), whereas no significant differences were observed for the other factors (p > 0.05). Compared to the YC group, the OC group demonstrated marked upregulation of TNFα (p < 0.001), NF-κB (p < 0.05) and IL-1β (p < 0.001) at the mRNA levels. Nevertheless, no significant difference in interleukin-6 (IL-6) and interleukin-10 (IL-10) levels (p > 0.05) was detected between the YC and OC groups (**Figures 3D, E**). Notably, the OR group exhibited substantial reductions in the mRNA levels of TNF-α (p < 0.05), NF-κB (p < 0.05), and IL-1β (p < 0.001) compared with OC group. In contrast, the mRNA levels of IL-6 (p < 0.05) and IL-10 (p < 0.05) were elevated. These findings indicated that resistance training modulates the mRNA levels of inflammatory factors, potentially alleviating age-related inflammatory responses in skeletal muscles.

### 3.4 Resistance training modulated the mTORC1-HIF-1α pathway and alleviated chronic inflammation

**Figure 4:**
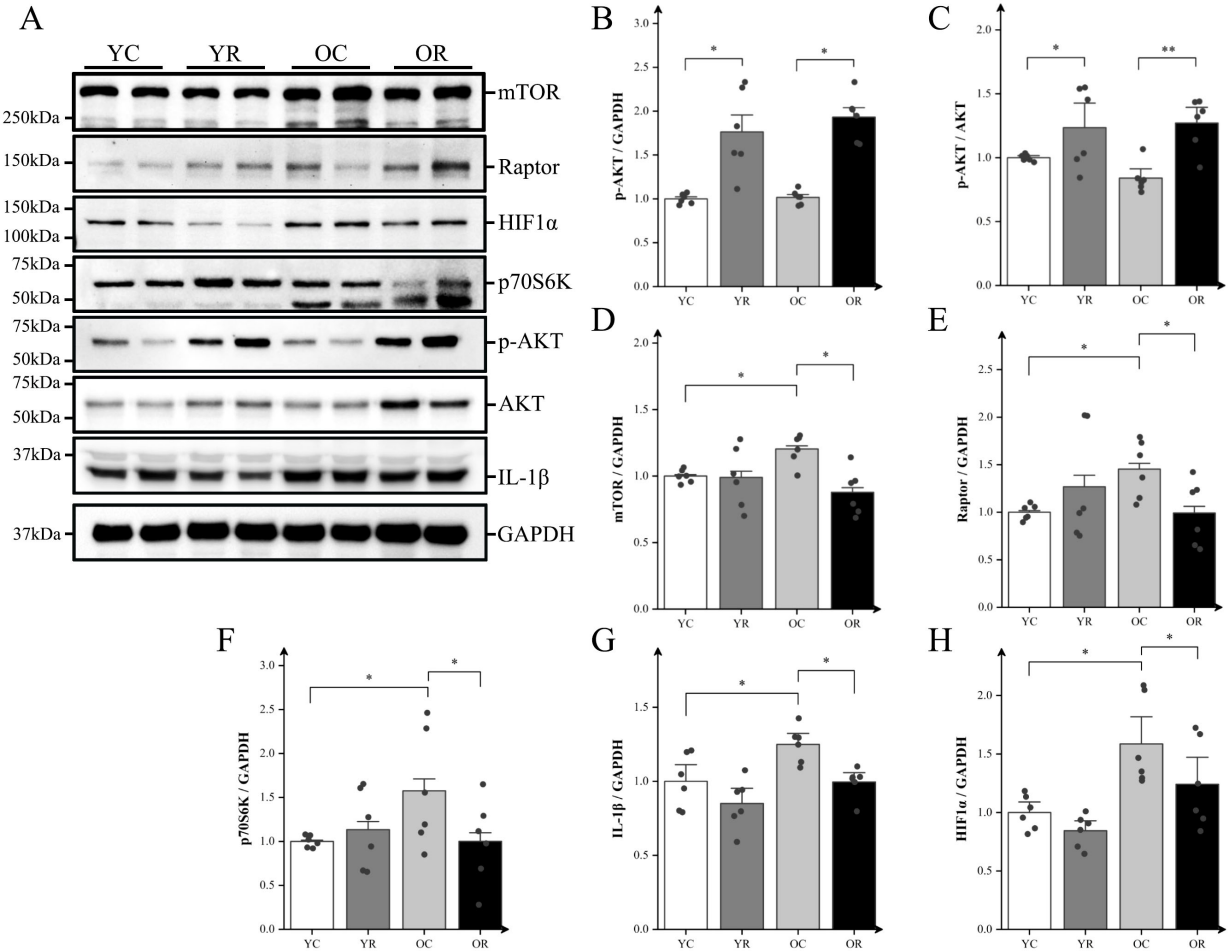
Resistance training regulated mTORC1-HIF-1α pathway and alleviated chronic inflammation. **(A)** Representative Western blot images of mTOR, Raptor, HIF-1α, p70S6K, p-AKT (Ser473), AKT, IL-1β, and the internal control GAPDH in the quadriceps muscle. **(B–H)** Protein expression levels of p-AKT (Ser473), p-AKT (Ser473)/AKT, mTOR, Raptor, HIF-1α, p70S6K, and IL-1β. All data are presented as means ± SEM (two-way ANOVA with Tukey *post hoc* test), n = 6 per group; *p < 0.05, **p < 0.01.

We analyzed the expression of inflammatory proteins using western blotting (**Figure 4A**). Compared with the YC group, resistance training significantly increased the expression of pho- protein kinase B (p-AKT) and the p-AKT/AKT ratio (p < 0.05; **Figures 4B, C**). The results demonstrated no significant differences in AKT phosphorylation status or p-AKT/AKT ratios were observed between OC and YC groups (p > 0.05). However, the OC group exhibited significantly elevated expressions of mTOR, Raptor, and p70S6K (p < 0.05; **Figures 4D–F**). Relative to the OC group, the OR group demonstrated enhanced AKT activation evidenced by increased phosphorylation (p < 0.05) and elevated p-AKT/AKT ratio (p < 0.01). Conversely, significant downregulation was observed in downstream signaling effectors: mTOR (p < 0.05), Raptor (p < 0.05), and p70S6K (p < 0.05). The inflammation-related proteins IL-1β and HIF-1α, which are regulated by mTORC1, were also examined. The expression of IL-1β and HIF-1α in the OC group was significantly upregulated compare to the YC group (p < 0.05; **Figures 4G, H**), whereas their expression in the OR group were significantly downregulated compared to the OC group (p < 0.05). In summary, our findings suggested that resistance training inhibits the mTORC1-HIF-1α pathway in the skeletal muscle of aging mice, indicating its potential role in regulating chronic inflammation in skeletal muscle.

### 3.5 Resistance training activated autophagy and altered mTORC1-AMPK pathway

**Figure 5:**
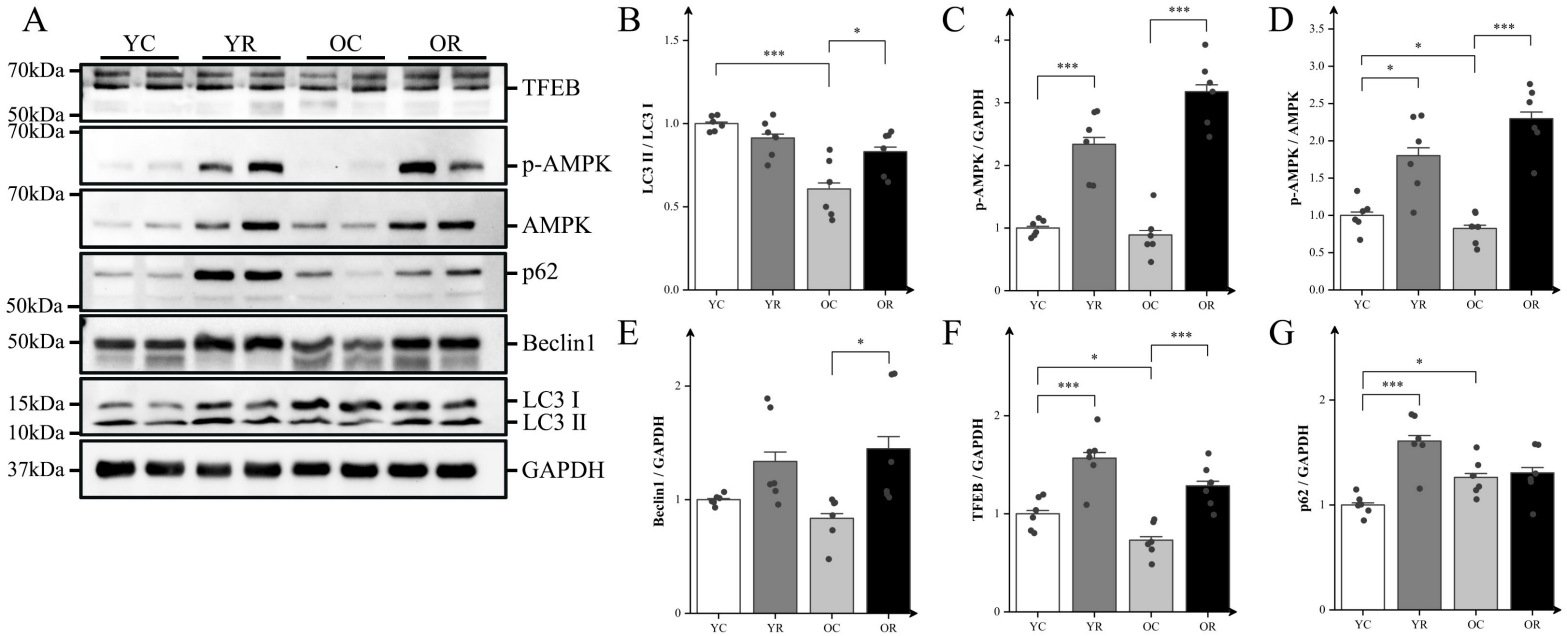
Resistance training activated autophagy and altered mTORC1-AMPK pathway. **(A)** Representative Western blot images of TFEB, p-AMPK (Thr172), AMPK, p62, Beclin1, LC3, and the internal control GAPDH in the quadriceps muscle. **(B–G)** Protein expression levels of LC3II/LC3I, p-AMPK (Thr172), p-AMPKα (Thr172)/AMPKα, Beclin1, TFEB, and p62. All data are presented as means ± SEM (two-way ANOVA with Tukey *post hoc* test), n = 6 per group. *p < 0.05, ***p < 0.001.

mTORC1 serves as a central regulator coordinating inflammatory responses and autophagy through its interaction with AMP-activated protein kinase (AMPK) and its downstream signaling proteins. Our results revealed that the microtubule-associated protein 1A/1B-light chain 3 (LC3) II/LC3 I ratio in the OC group was significantly lower compared with that in the YC group (p < 0.001; **Figure 5B**). Conversely, the OR group demonstrated a significantly higher LC3 II/LC3 I compare to the OC group (p < 0.05), suggesting that resistance training may alleviate age-related decline in autophagy. To validate this finding further, we analyzed the expression of autophagy-related proteins (**Figures 5C–G**). Our results showed that, compared with the YC group, resistance training significantly increased the protein expression of p-AMPK (p < 0.001), TFEB (p < 0.001), and p62 (p < 0.001), as well as the p-AMPK/AMPK ratio (p < 0.05). In contrast to the YC group, the OC group exhibited a statistically significant decrease in the p-AMPK/AMPK ratio (p < 0.05), transcriptional factor EB (TFEB) (p < 0.05), and p62 (p < 0.05), while no statistically significant differences were detected for p-AMPK and BCL-2-interacting protein 1 (Beclin1) (p > 0.05). In contrast, the OR group demonstrated significant increases in p-AMPK (p < 0.001), Beclin1 (p < 0.05), TFEB (p < 0.001), and the p-AMPK/AMPK ratio (p < 0.001) compared to the OC group. These findings suggested that resistance training can enhance autophagy during age-related muscle atrophy.

## 4 Discussion

This study evaluated the potential of resistance training in mitigating age-related muscle atrophy. Resistance training significantly increased wet muscle weight (GAS and Quad), CSA, maximal load, and grip strength in aged mice compared to YC group. In contrast, the soleus and tibialis anterior showed no significant hypertrophy, likely due to differences in fiber type composition, functional roles, and recruitment patterns. The gastrocnemius and quadriceps, rich in type II fibers, are more responsive to hypertrophic stimuli, whereas the soleus, dominated by type I fibers, is less sensitive to resistance-induced growth (40). Moreover, the training protocol likely provided a greater mechanical load to GAS and Quad than SOL and TA, leading to muscle-specific adaptations (41, 42). Following 8-week resistance training, inflammation was significantly mitigated, as evidenced by enhanced macrophage polarization towards an anti-inflammatory phenotype and reduced mRNA levels of inflammatory cytokines. Moreover, the protein expression of the mTORC1-HIF-1α pathway was diminished. Furthermore, resistance training promoted autophagy in aged muscles through the mTORC1-AMPK pathway.

As evidenced by recent studies, macrophage polarization is involved in host defense and inflammatory pathology (43, 44). iNOS and CD206 can reflect the polarization of macrophages, which has been proven in many experiments such as immunohistochemistry (45–48). M1 and M2 macrophages play crucial roles in tissue immunity and repair through the secretion of cytokines, including IL-1, IL-6 and IL-10 (49, 50), which further drive the process of macrophage polarization (51). Research has shown that in aging skeletal muscle, macrophages tend to polarize towards a pro-inflammatory phenotype, and the decline in muscle mass, strength, and physical health is linked to increased levels of pro-inflammatory cytokines (52–54). Therefore, modulation of macrophage polarization and cytokine secretion may be key to preventing and treating skeletal muscle aging. Previous research has shown that resistance training prevents the rise in circulating TNF-α levels in aged mice and decreases the mRNA expression of inflammatory cytokines (TNF-α and IL-1β) in skeletal muscle (18). Our study corroborates these findings, demonstrated that resistance training promoted macrophage polarization to the M2 phenotype, decreased the mRNA levels of TNF-α, NF-κB, and IL-1β, and elevated the mRNA levels of IL-6 and IL-10 in aged muscle. Prior research demonstrated that IL-6 had both pro-inflammatory and anti-inflammatory properties. Conventional perspectives have predominantly characterized interleukin-6 (IL-6) as a pro-inflammatory cytokine in skeletal muscle (55). On the contrary, during muscle contraction, IL-6 is produced and released into circulation by muscle tissue, suggesting that resistance training may increase skeletal muscle IL-6 secretion to enhance its anti-inflammatory effects (56). These findings indicated that resistance training may help mitigate inflammation in aging skeletal muscle.

The mTORC1 complex is involved in the integration of various signals to regulate various cellular processes in mammals (57). Studies have indicated that the activation of mTORC1 signal transduction triggers inflammation by inducing NF-κB activation and the production of disease-specific cytokines and chemokines (58). Conversely, Raptor knockout down suppressed mTORC1 activity and improved autophagy, oxidative stress, and inflammation (59). Prior research has consistently demonstrated that mTORC1 is hyperactivated in aged skeletal muscles, contributing to muscle atrophy (19, 60). Resistance training has been reported to mitigate age-related chronic inflammation in skeletal muscle by reducing the expression of cytokines such as TNFα and IL-1β (18, 60). Our 8-week training protocol alleviated chronic inflammation and reduced the expression of mTORC1, IL-1β and HIF-1α, suggesting that resistance training helps mitigate inflammation in aging skeletal muscles. However, while AKT is a positive regulator of mTORC1, our results showed that resistance training activated upstream AKT while inhibiting mTORC1 activity. Previous studies have confirmed that Sestrin2 negatively regulates mTORC1 activity, and that resistance training can alleviate skeletal muscle atrophy by up-regulating Sestrin2 expression (33, 61). We hypothesized that alternative pathways may contribute to the negative regulation of mTORC1 by resistance training; however, further research is required to confirm this assumption.

mTORC1 is involved in both inflammation and autophagy regulation through multiple pathways, including the mTORC1-AMPK and mTORC1-TFEB signaling pathways (62–65). Studies have shown that autophagy decreases with age. Wilhelm et al. investigated autophagic activity in C. elegans by employing fluorescence-labeled Immunoglobulin G 1 (LGG-1), revealing suppressed late-stage autophagy in aging worms (66). Additionally, Carnio et al. assessed autophagy marker expression, including LC3 and Atg7, in murine and human muscle tissue, demonstrating diminished autophagy activity in both species (67). Our findings corroborate previous reports showing a lower LC3II/LC3I ratio in aged mice, indicating impaired autophagy in aging skeletal muscle. Activated autophagy inhibits mTORC1 and reduces the expression of pro-inflammatory factors (68). In our study, resistance training reduced mTORC1 expression, increased AMPK activity, and elevated the protein expressions of Beclin1 and TFEB, indicating a positive effect on autophagy. These findings are consistent with those reported by Wang et al., who demonstrated that prolonged aerobic and resistance training could modulate autophagy-related proteins, thereby promoting skeletal muscle autophagy and mitigating muscle mass loss (68). Therefore, mTORC1 pathway regulation might mitigate inflammation and improve autophagy in aged skeletal muscle. While both the mTORC1–HIF-1α and mTORC1–AMPK pathways are modulated by mTORC1, increasing evidence indicates functional cross-talk between AMPK and HIF-1α signaling. Specifically, AMPK activation has been shown to inhibit HIF-1α accumulation by promoting its proteasomal degradation and reducing transcription of HIF-1α target genes, including those involved in glycolysis and inflammatory responses (69, 70). Furthermore, AMPK negatively regulates mTORC1 activity, thereby indirectly suppressing HIF-1α expression through upstream inhibition (71, 72). These interactions suggest that AMPK may serve as a critical modulator in the resistance exercise-mediated suppression of the mTORC1–HIF-1α axis, contributing to the attenuation of inflammation and autophagy in aged skeletal muscle (73).

It is necessary to acknowledge the limitations of this work. Firstly, potential confounding factors, such as diet and individual animal variability, were considered in this study. To minimize these influences, a standardized diet was provided to all animals throughout the experiment, and appropriate sample sizes were used to account for individual variability, thereby ensuring the robustness of our findings. Secondly, although immunofluorescence can quantify the expression of iNOS and CD206, its limitation is that it does not accurately distinguish whether they are derived from skeletal muscle or macrophages. In future studies, flow cytometry or single-cell RNA sequencing could be employed to more accurately delineate macrophage dynamics during the process of training adaptation.

This study highlighted the crucial role of mTORC1 in mediating the beneficial effects of resistance training in aging skeletal muscles. Specifically, it highlights the impact on reducing chronic inflammation, enhancing autophagy, and improving the overall muscle quality. Our findings may have important implications for human applications, particularly in resistance training for the elderly. The results suggest that resistance exercise could play a key role in mitigating age-related muscle degeneration and sarcopenia. These insights could inform clinical guidelines and resistance training protocols aimed at improving muscle strength and functionality in older adults, thereby enhancing their overall quality of life. Future research should investigate the impact of varying intensities of resistance training on inflammation- and autophagy-related proteins to make training protocols for mitigating age-related muscle atrophy.

## Data Availability

The datasets presented in this study can be found in online repositories. The names of the repository/repositories and accession number(s) can be found in the article/**Supplementary Material**.
