# Supplementary material for: Resistance training alleviates muscle atrophy and muscle dysfunction by reducing inflammation and regulating compromised autophagy in aged skeletal muscle
Source: Front Immunol. 2025 Jun 3;16:1597222. doi: 10.3389/fimmu.2025.1597222 (PMC12170331; doi:10.3389/fimmu.2025.1597222)
Supplement: Supplementary file 1 [file DataSheet1.pdf]

Supplementary Information

Wet Muscle Weight

Number	SOL	GAS	TA	QUAD
YC1	0.0118	0.1532	0.08335	0.1703
YC2	0.0197	0.1634	0.06785	0.239
YC3	0.0267	0.1428	0.0748	0.21215
YC4	0.0213	0.1631	0.091	0.1549
YC5	0.0147	0.14385	0.07355	0.25445
YC6	0.0185	0.153	0.0824	0.22515
YR1	0.0191	0.16165	0.08155	0.23375
YR2	0.0182	0.15195	0.08195	0.20885
YR3	0.0192	0.14295	0.08065	0.22655
YR4	0.0247	0.18285	0.08625	0.20895
YR5	0.0196	0.1452	0.0767	0.2305
YR6	0.0204	0.1488	0.08735	0.2351
OC1	0.0118	0.13815	0.04955	0.19495
OC2	0.0102	0.1427	0.05025	0.20205
OC3	0.01035	0.13855	0.05095	0.16935
OC4	0.00995	0.1394	0.05115	0.20655
OC5	0.01285	0.134	0.0518	0.1857
OC6	0.0098	0.14625	0.0518	0.2015
OR1	0.01155	0.1656	0.05575	0.2168
OR2	0.0094	0.1727	0.0535	0.2395
OR3	0.0111	0.1798	0.0521	0.23125
OR4	0.00975	0.1602	0.0553	0.2551
OR5	0.00855	0.17075	0.0579	0.219
OR6	0.0103	0.1644	0.0554	0.1843

Muscle Function

MGS（Before）																			
Group	Number	Weight	1	2	3	4	5	6	7	8	9	10	11	12	13	14	15	g	
Con1	1	38	20	30	40	50	60	70	80xxx	75xxx	73xxx	72xx						72	
	2	37	20	30	40	50	60	70	80	90xxx	85	87xxx						85	
	3	39.5	20	30	40	50	60	70	80xxx	75xxx	73							73	
	4	32	20	30	40	50	60	70	80xxx	75	77x	79xx						79	
	5	38	20	30	40	50	60	70	80	90xxx	85xxx	83xxx	82xx					82	
Con2	1	39	20	30	40	50	60	70	80	90xxx	85xxx	83x						83	
	2	39.5	20	30	40	50	60	70	80xxx	75	77x	78x						78	
	3	37	20	30	40	50	60	70	80x	90xxx	85xxx	83xxx	82x					82	
	4（Dead）	41																	
	5	36.5	20	30	40	50	60	70	80xxx	75	77xxx	76x						76	
AEA1	1	38	20	30	40	50	60	70	80xxx	75x	77xxx	76xx						76	
	2	36	20	30	40	50	60	70	80xxx	75xxx	73	74x						74	
	3	33.5	20	30	40	50	60	70	80	90xxx	85xxx	83x						83	
	4	39	20	30	40	50	60	70	80	90xxx	85	87x	88x	89xxx				88	
	5	42.5	20	30	40	50	60	70	80	75xxx	73	74x						74	
AEA2	1	38	20	30	40	50	60	70	80	90xxx	85xxx	83x						83	
	2	38.5	20	30	40	50	60	70	80xxx	75x	77x	78xxx						77	
	3	32.5	20	30	40	50	60	70	80	75xxx	73	74x						74	
	4	31.5	20	30	40	50	60	70	80xxx	75	77xxx	76x						76	
	5	37	20	30	40	50	60	70	80	90xxx	85xxx	83x	84xxx					83	
REA1	1	35	20	30	40	50	60	70	80	90xxx	85xxx	83x	84x					84	
	2	39.5	20	30	40	50	60	70	80	90xxx	85xxx	83xxx	82xx					82	
	3	39.5	20	30	40	50	60	70	80	90xxx	85	87x	88xxx					87	
	4（Dead）																		
	5	39	20	30	40	50	60	70	80	90xxx	85xxx	83x	84xxx					83	
REA2	1	40	20	30	40	50	60	70	80x	90xxx	85xxx	83x	84x					84	
	2	26	20	30	40	50	60	70	80xxx	75xxx	73x	74xxx						73	
	3	36	20	30	40	50	60	70	80xxx	75xxx	73	74x						74	
	4	38.5	20	30	40	50	60	70	80xxx	75x	77x	78xxx						77	
	5	39	20	30	40	50	60	70	80xxx	75	77xxx	76xxx						75	

Muscle Function

MDS(After)																					
Group	Number	Weight	1	2	3	4	5	6	7	8	9	10	11	12	13	14	15	16	17	g	
Con1	1	38	30	40	50	60	70	80	75xxx	73xxx	72xxx	71x								71	
	2	37	30	40	50	60	70	80	90xxx	85xxx	83x	84xxx								83	
	3	39.5	30	40	50	60	70	80x	90xxx	85xxx	83xxx	82xxx	81xxx							80	
	4	32	30	40	50	60	70	80xxx	75	77xxx	76x									76	
	5	38	30	40	50	60	70	80	90xxx	85xxx	83x	84x								84	
Con2	1	39	30	40	50	60	70	80x	90xxx	85xxx	83xxx	82xxx	81xxx							80	
	2	39.5	30	40	50	60	70	80xxx	75xxx	73xxx	72xxx	71xxx								70	
	3 (Dead)																				
	4(dead)																				
	5	36.5	30	40	50	60	70	80	90xxx	85xxx	83x	84xxx								83	
AEA1	1	38	30	40	50	60	70	80	90xxx	85	87x	88xxx								87	
	2	36	30	40	50	60	70	80	90	100xxx	95xxx	93xxx	92xxx	91xxx						90	
	3	33.5	30	40	50	60	70	80	90	100xxx	95xxx	93xxx	92x							92	
	4	39	30	40	50	60	70	80	90	100	110xxx	105xxx	103xxx	102xxx	101x					101	
	5	42.5	30	40	50	60	70	80	90	100xxx	95xxx	93xx	94xxx							93	
AEA2	1	38	30	40	50	60	70	80	90	100xxx	95	97x	98x	99xxx						98	
	2	38.5	30	40	50	60	70	80	90	100	110xxx	105xxx	103xxx	102xxx	101xxx					100	
	3	32.5	30	40	50	60	70	80	90	100	110xxx	105xxx	103xxx	102x						102	
	4	32.5	30	40	50	60	70	80	90	100	110xxx	105xxx	103xx	104xxx						103	
	5	37	30	40	50	60	70	80	90	100	110xxx	105xxx	103xx	104xx						104	
REA1	1	35	30	40	50	60	70	80	90	100	110	120	130	140	150xxx	145xxx	143xxx	142x		142	
	2	39.5	30	40	50	60	70	80	90	100	110	120	130	140	150xxx	145xxx	143xxx	142x	141xxx	140	
	3 (Dead)																				
	4 (Dead)																				
	5	39	30	40	50	60	70	80	90	100	110	120	130	140	150xxx	145xxx	143xxx	142x		142	
REA2	1	40	30	40	50	60	70	80	90	100	110	120	130xxx	125	127x	128x	129xxx			128	
	2	26	30	40	50	60	70	80	90	100	110	120	130	140xxx	135xxx	133xxx	132xxx	131xxx		130	
	3	36	30	40	50	60	70	80	90	100	110	120	130	140xxx	135	137x	138xx	139xx		139	
	4	38.5	30	40	50	60	70	80	90	100	110	120	130xxx	125	127xxxx	126xxx				125	
	5	39	30	40	50	60	70	80	90	100	110	120	130xxx	125	127x	128xxx				127	

Muscle Function

ML(Before)

Group	Number	Weight	1	2	3	4	5	6	7	8	9	10	11	12	13	14	15	G
REA1	1	30.5	17.5	24.5	31.5XXX	25.5	26	27xxx										26
	2	33.5	19.75	27.65	35.55	31.6	32	33xxx										32
	3		19.75	27.65	35.55xxx	31.6	32	33	34xxx									32
	4（Dead）																	
	5	31.5	19.5	27.65	31.5	35.5	39xxx	36xxx	35.5									35.5
REA2	1	34.5	20	28	32	36	40xxx	37	38	39xxx								38
	2	25.5	13	18.5	20.5	23.5xxx	21	22	23xxx									22
	3	31	18	25.5	28.5	32.5	36	39.5xxx	37xxx									36
	4	33	19.25	26.5	34.5	38.5	39.5xxx	38.5										38.5
	5	33	19.5	27.5	35.5	39xxx	36	37	38xxx									37

ML(After)

Group	Number	Weight	最大负重（干预前）	1（100%）	2（150%）	3（160）%	4（170%）	5（180%）	6（190%）	7（200%）	8（210%）	9（220%）	10（230%）	11（240%）	12（250%）	13（260%）	14(270%)	15（280%）	G
REA1	1	30.5	26	26	39	41.6	44.2	46.8	49.4	52	54.6	57.2	59.8	62.4	65	67.6	70.2	72.8	72.8(280%)
	2	33.5	32	32	48	51.2	54.4	57.6	60.8	64	67.2	70.4	73.6	76.8	80	83.2	86.4	89.6	73.6(230%)
	3（Dead）																		
	4（Dead）																		
	5	31.5	35.5	35.5	53.25	56.8	60.35	63.9	67.45	71	74.55	78.1	81.65	85.2	88.75	92.3	95.85	99.4	83.425（235%）
REA2	1	34.5	38	38	57	60.8	64.6	68.4	72.2	76	79.8	83.6	87.4	91.2	95	98.8	102.6	106.4	72.2(190%)
	2	25.5	22	22	33	35.2	37.4	39.6	41.8	44	46.2	48.4	50.6	52.8	55	57.2	59.4	61.6	55(250%)
	3	31	36	36	54	57.6	61.2	64.8	68.4	72	75.6	79.2	82.8	86.4	90	93.6	97.2	100.8	72(200%)
	4	33	38.5	38.5	57.75	61.6	65.45	69.3	73.15	77	80.85	84.7	88.55	92.4	96.25	100.1	103.95	107.8	73.15(185%)
	5	33	37	37	55.5	59.2	62.9	66.6	70.3	74	77.7	81.4	85.1	88.8	92.5	96.2	99.9	103.6	88.8(240%)

H&E Staining

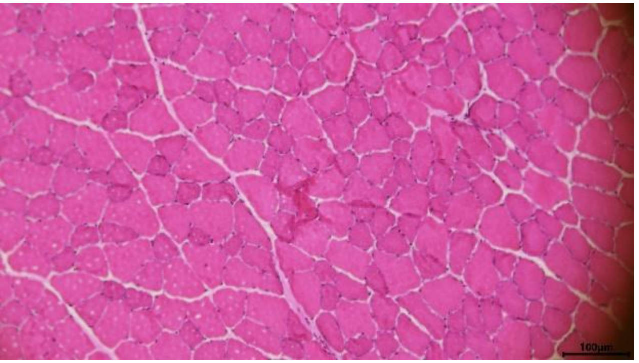
YC 20

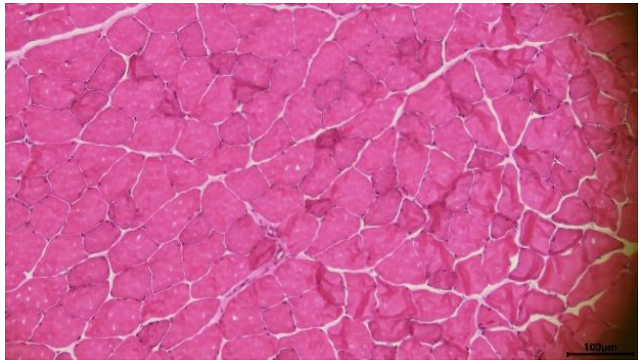
YR 20

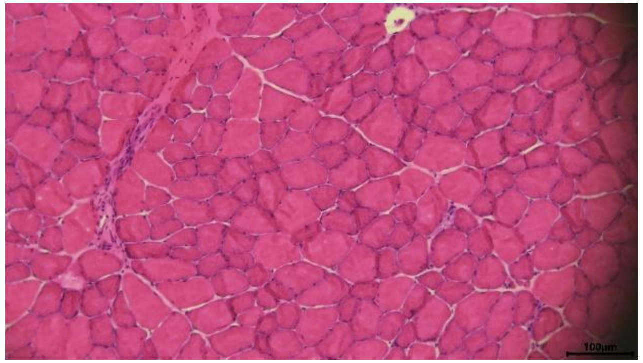
OC 20

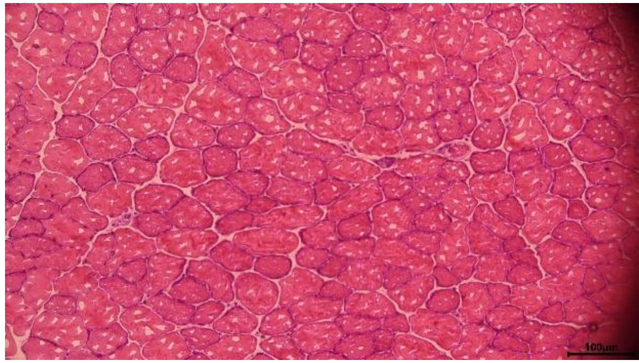
OR 20

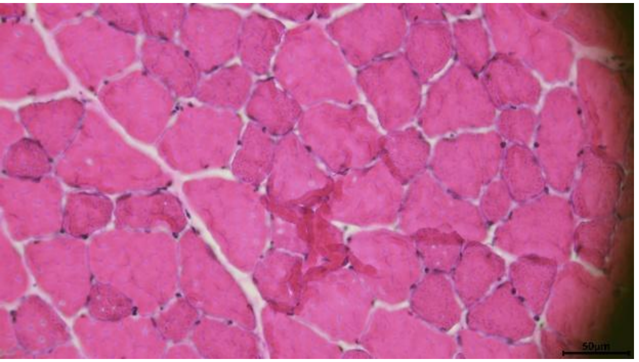
YC 40

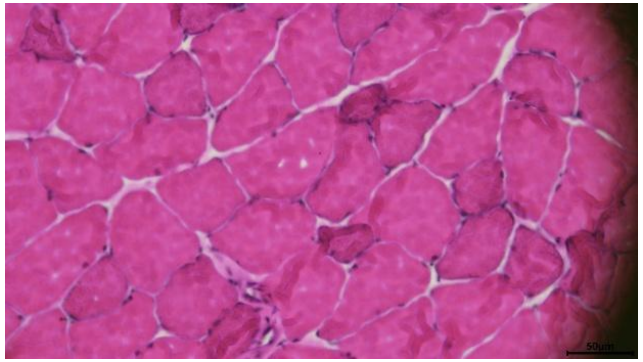
YR 40

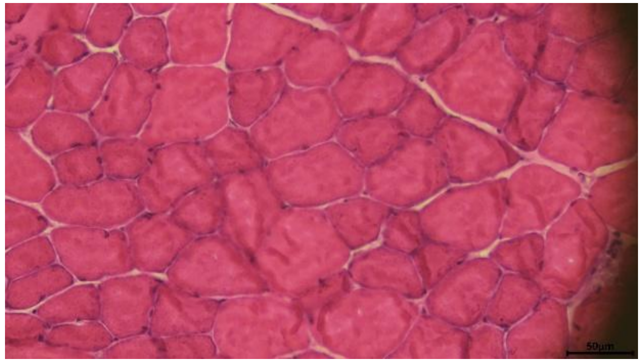
OC 40

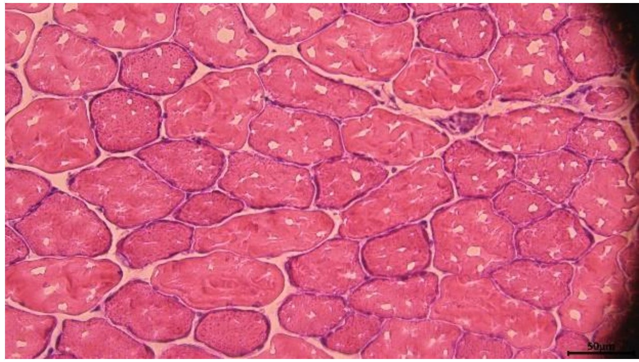
OR 40

I F

INOS-YC20 (INOS, DAPI and Merge)

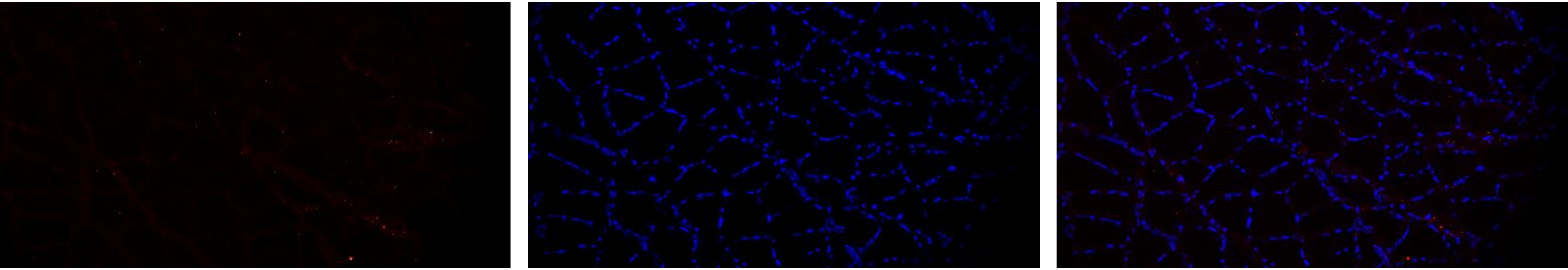

INOS-YC40 (INOS, DAPI and Merge)

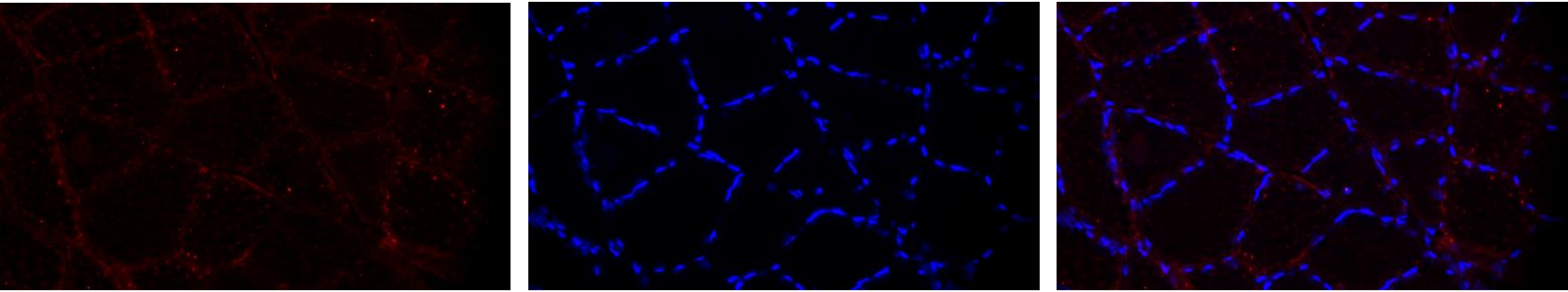

INOS-YR20 (INOS, DAPI and Merge)

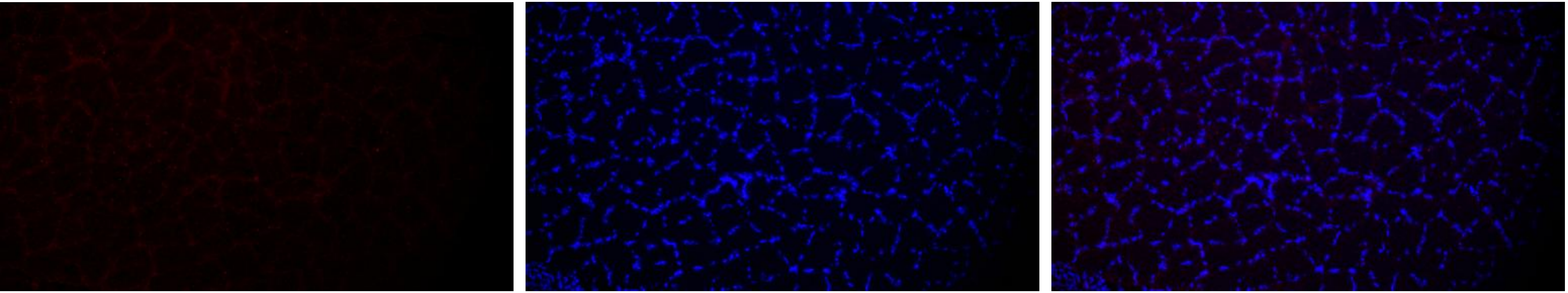

INOS-YR40 (INOS, DAPI and Merge)

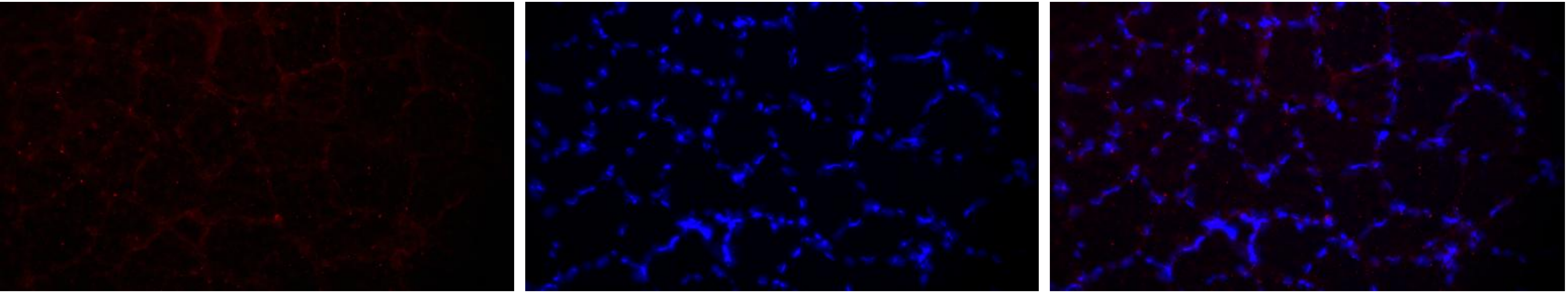

INOS-OC20 (INOS, DAPI and Merge)

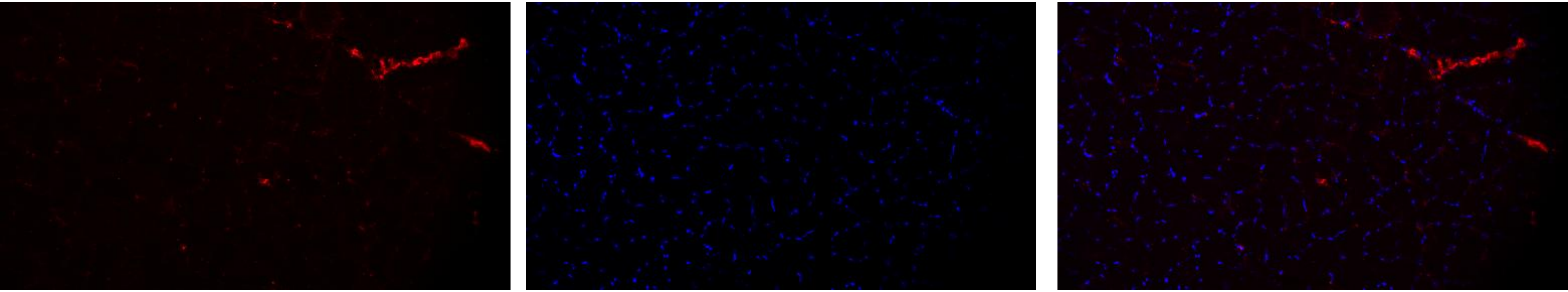

INOS-OC40 (INOS, DAPI and Merge)

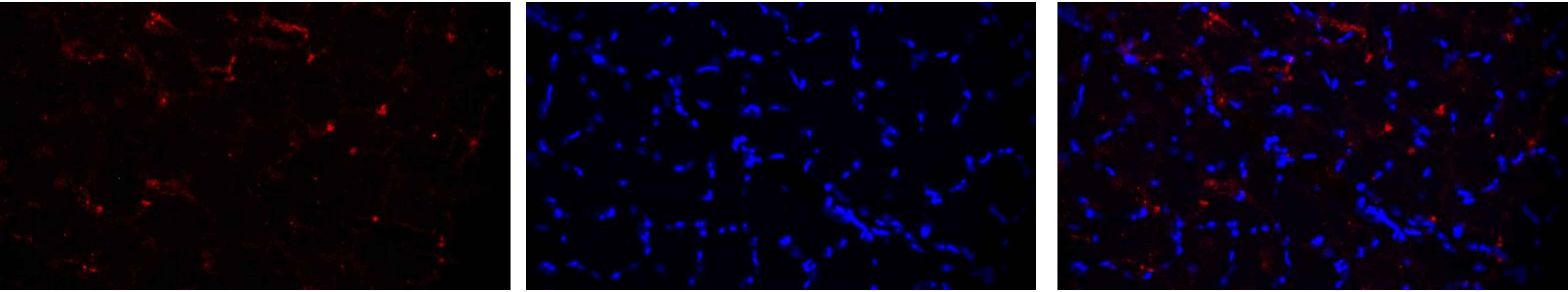

INOS-OR20 (INOS, DAPI and Merge)

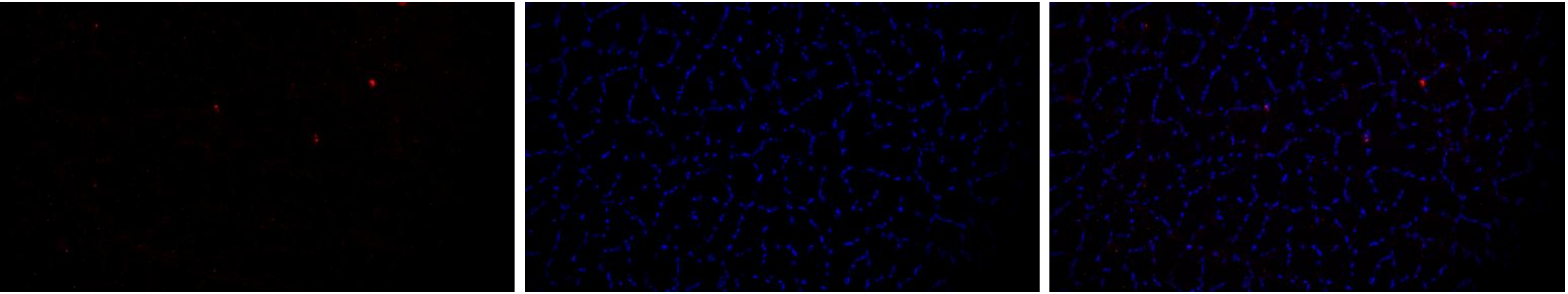

INOS-OR40 (INOS, DAPI and Merge)

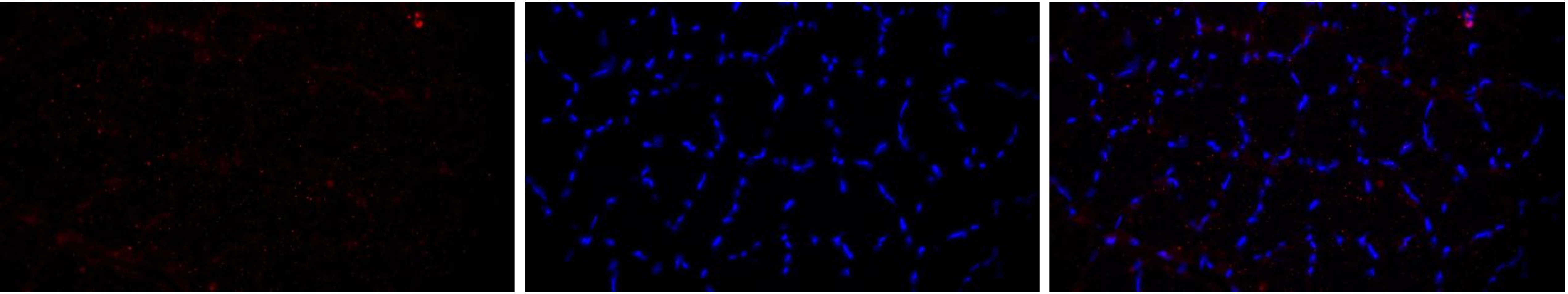

CD206-YC20 (CD206, DAPI and Merge)

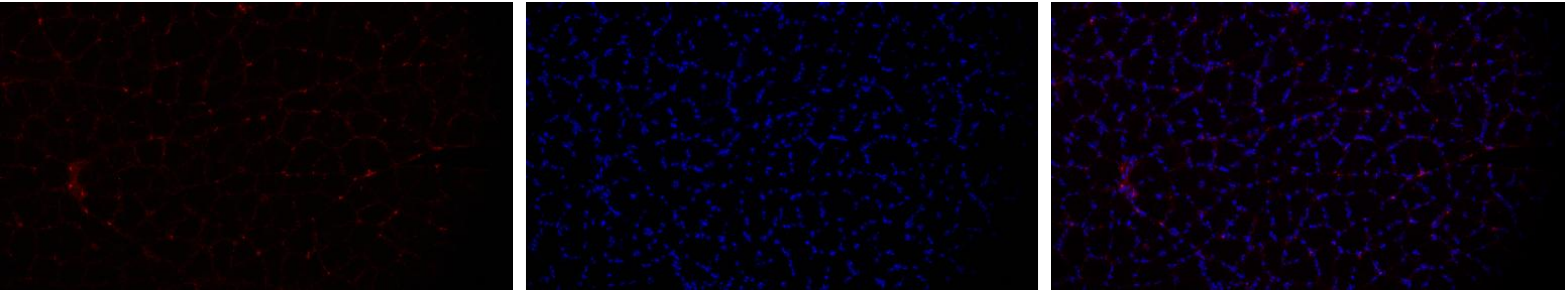

CD206-YC40 (CD206, DAPI and Merge)

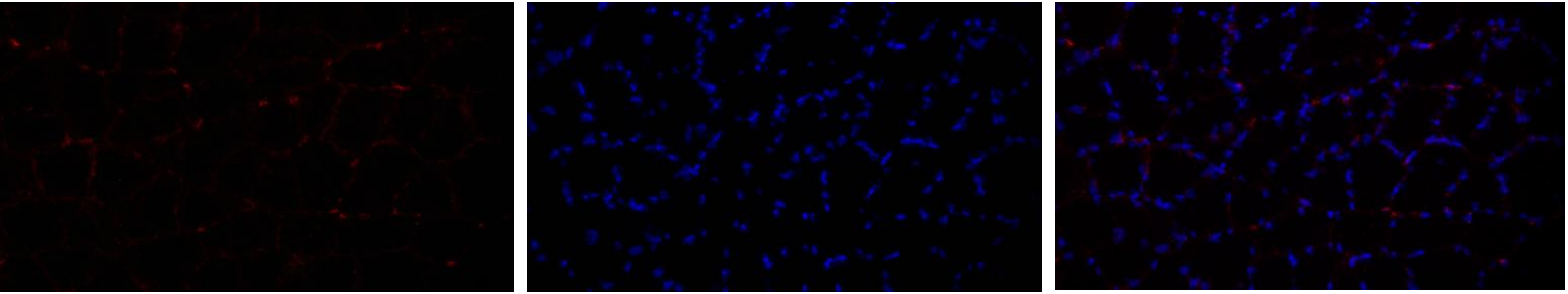

CD206-YR20 (CD206, DAPI and Merge)

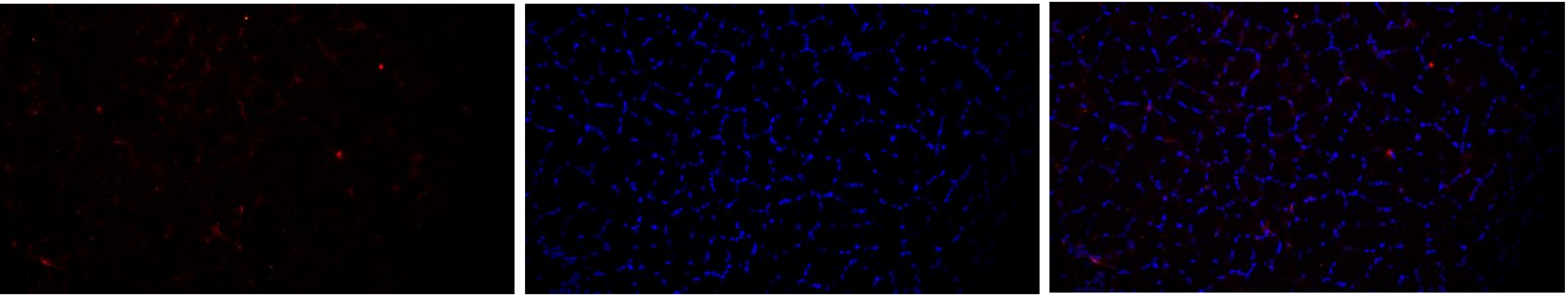

CD206-YR40 (CD206, DAPI and Merge)

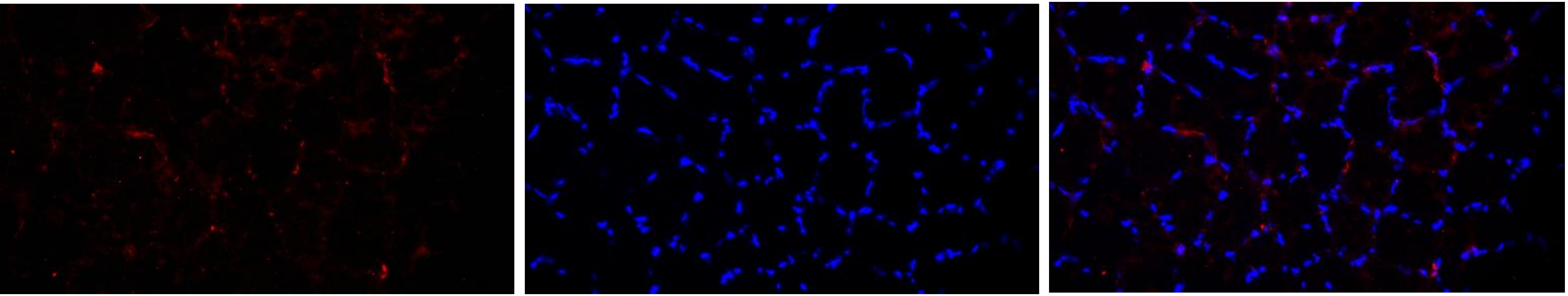

CD206-OC20 (CD206, DAPI and Merge)

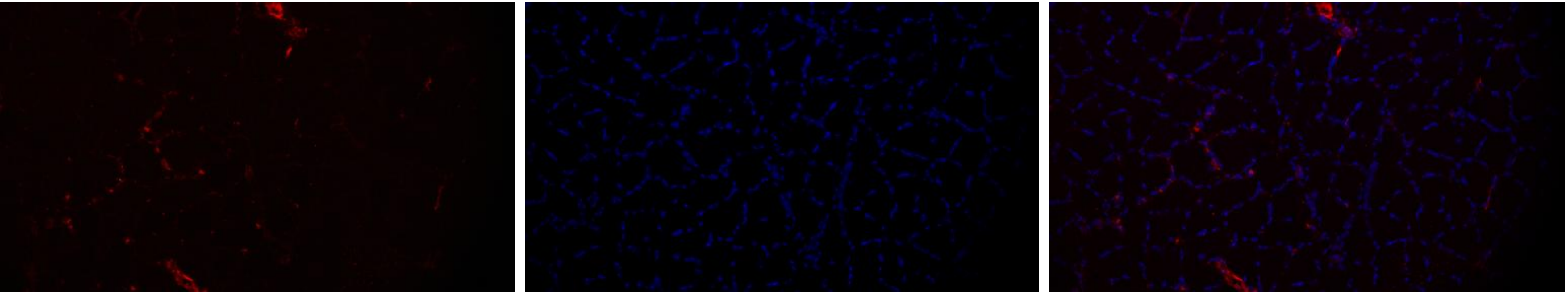

CD206-OC40 (CD206, DAPI and Merge)

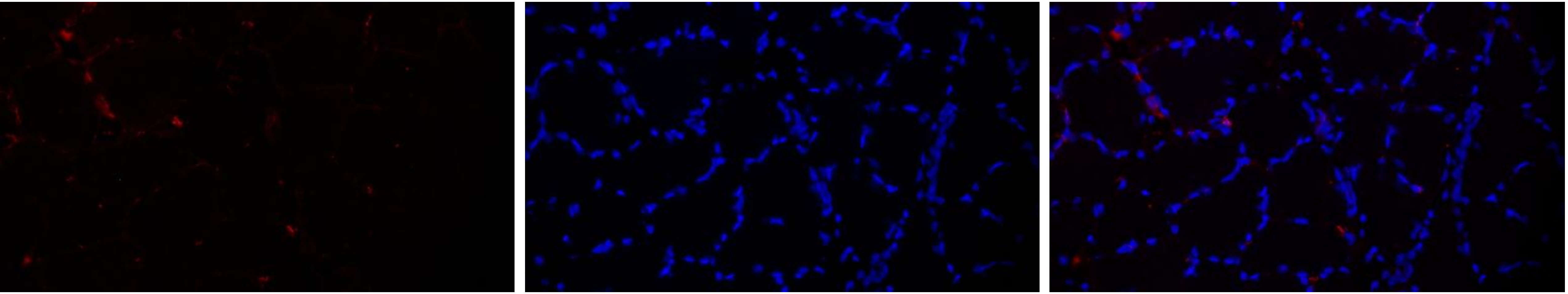

CD206-OR20 (CD206, DAPI and Merge)

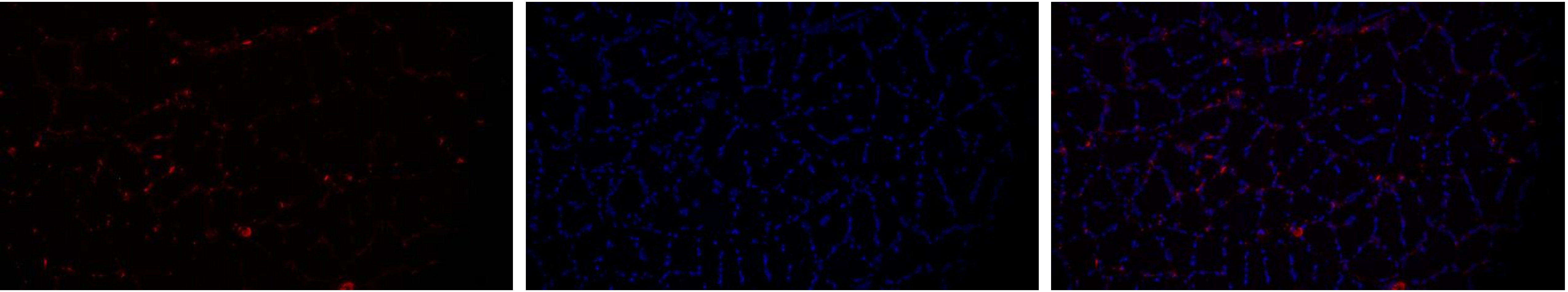

CD206-OR40 (CD206, DAPI and Merge)

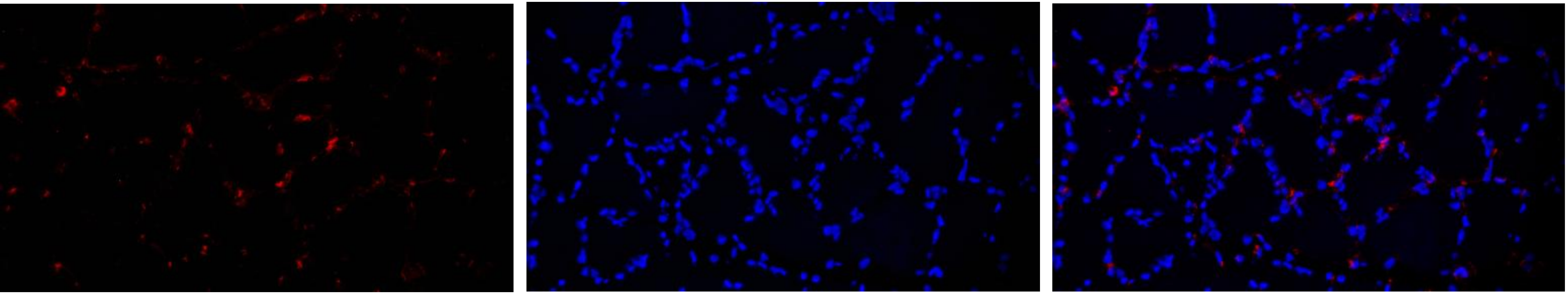

Westren Blot——AKT

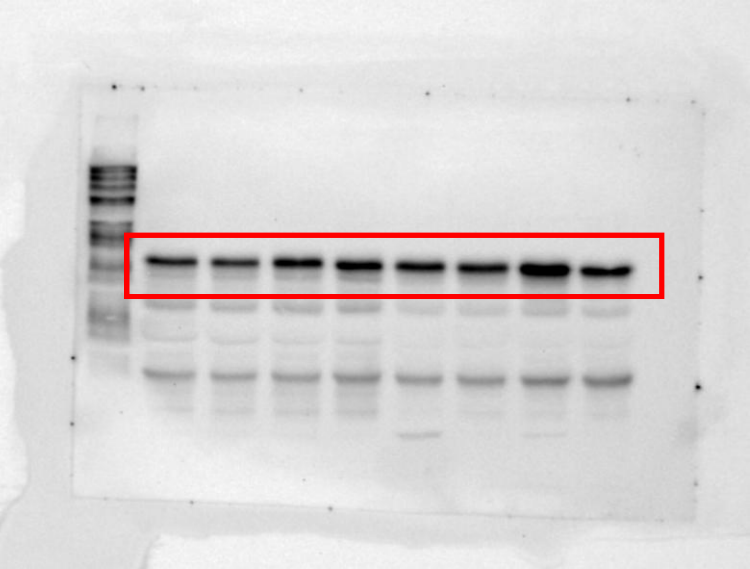

1-2

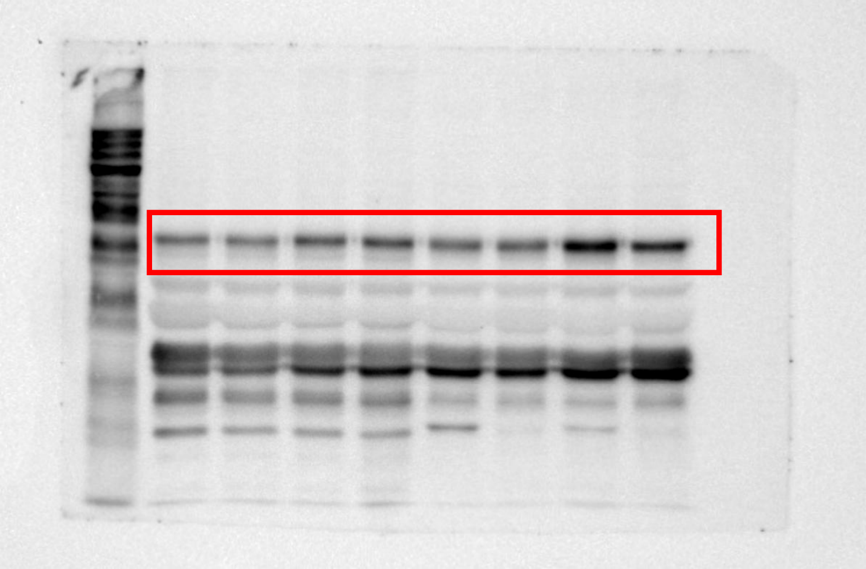

3-4

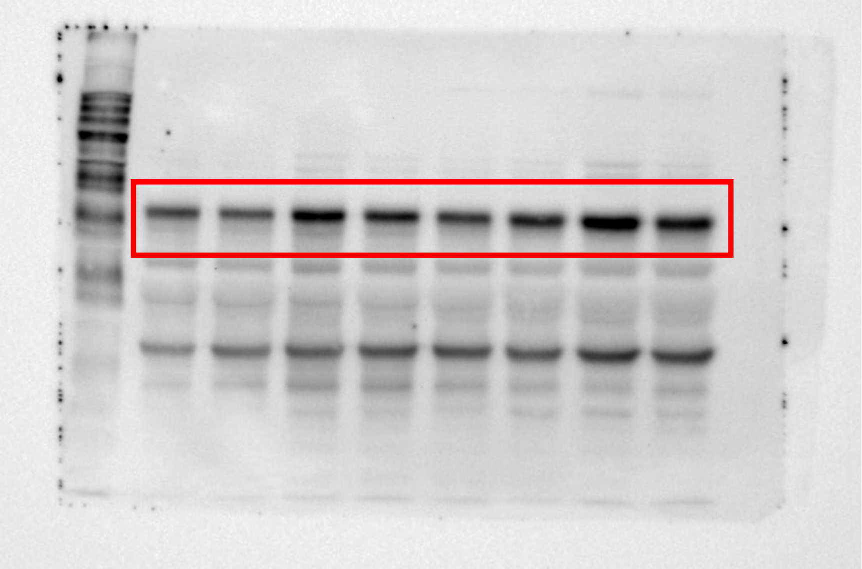

5-6

AKT（GAPDH）

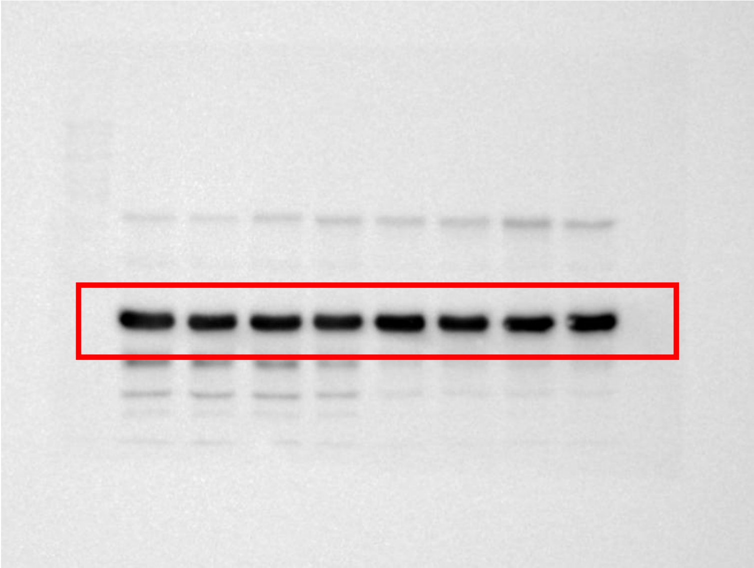

1-2

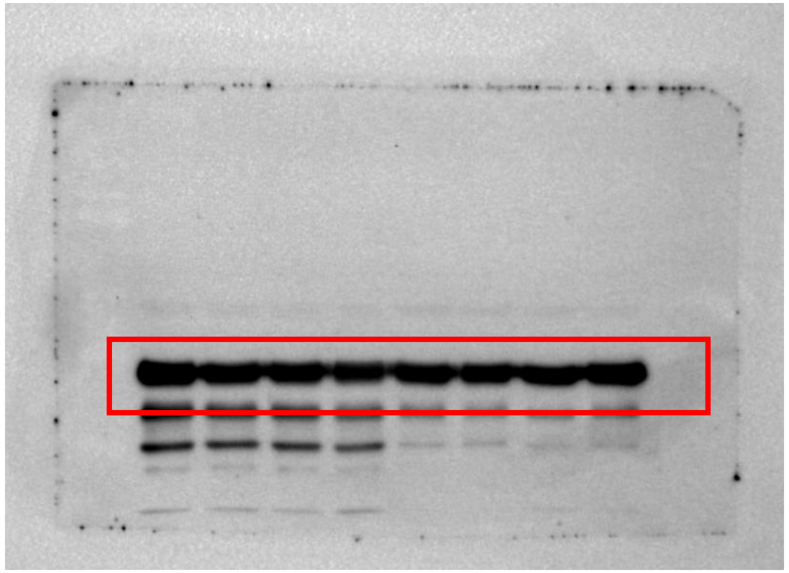

3-4

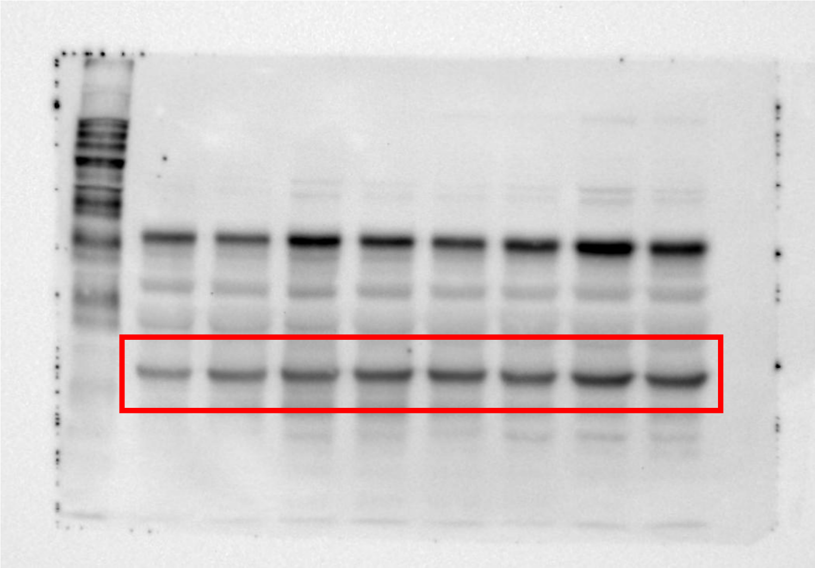

5-6

P-AKT

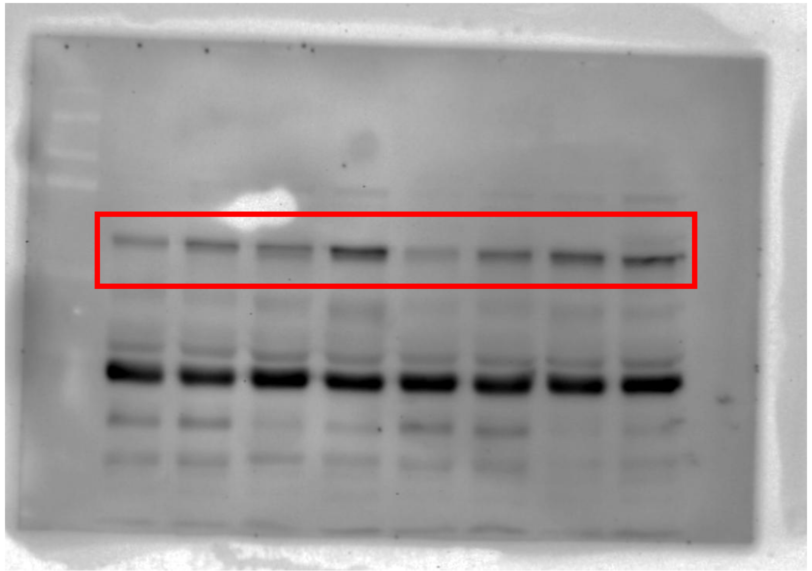

1-2

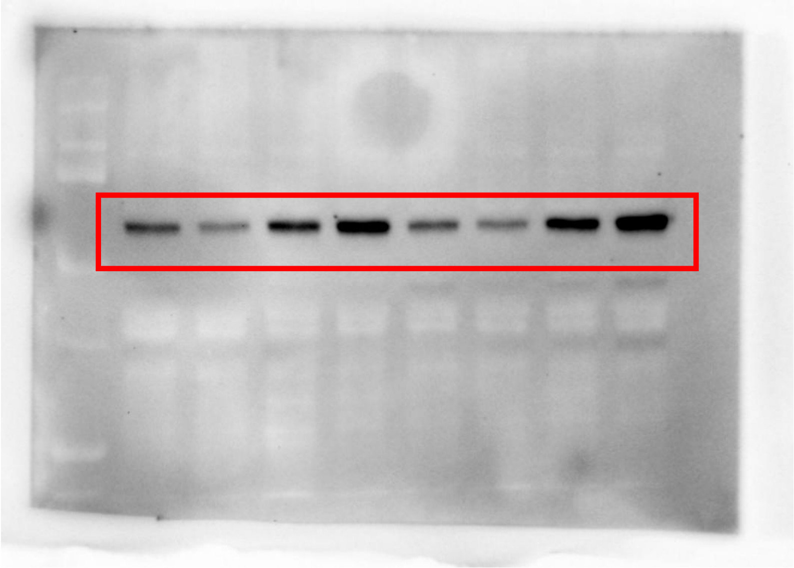

3-4

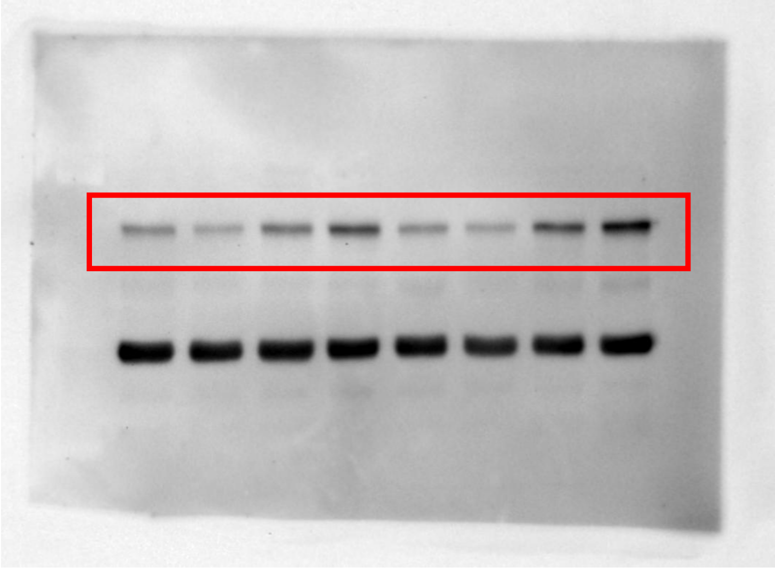

5-6

P-AKT(GAPDH)

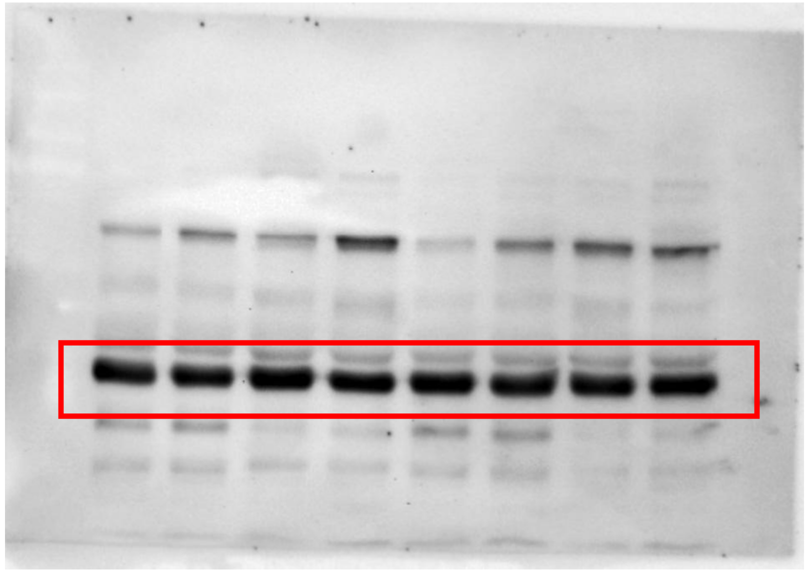

1-2

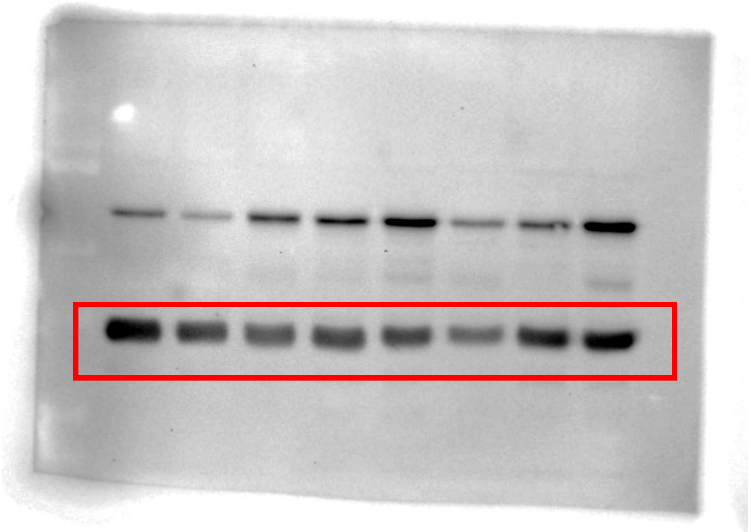

3-4

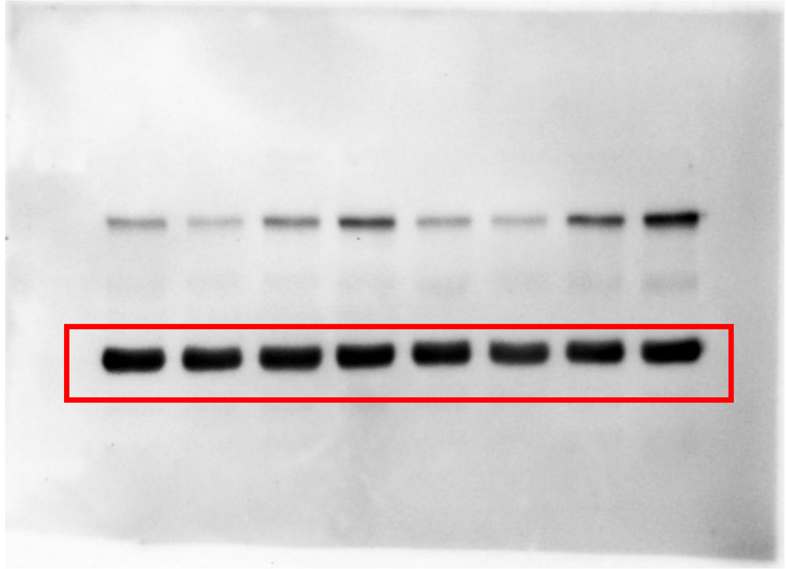

5-6

AMPK

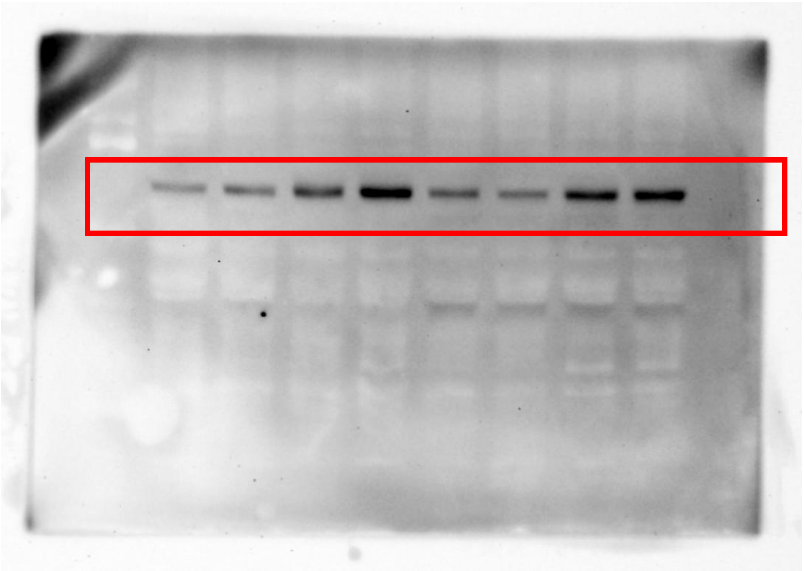

1-2

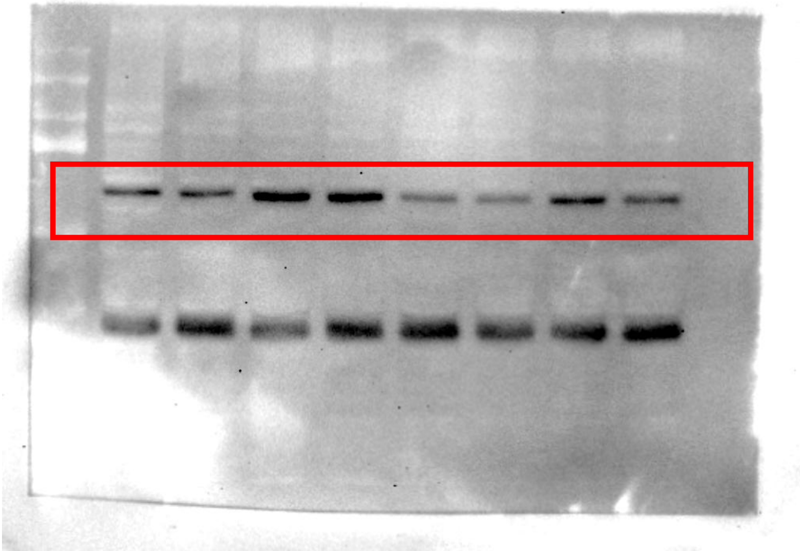

3-4

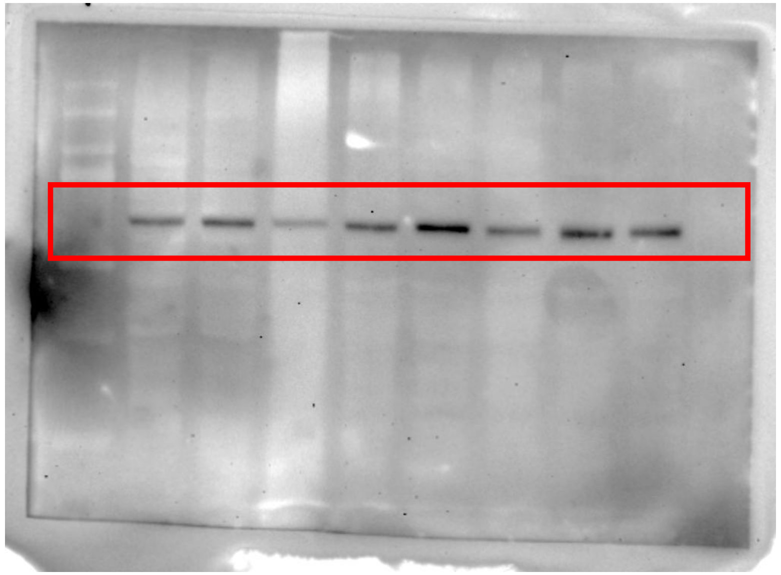

5-6

AMPK(GAPDH)

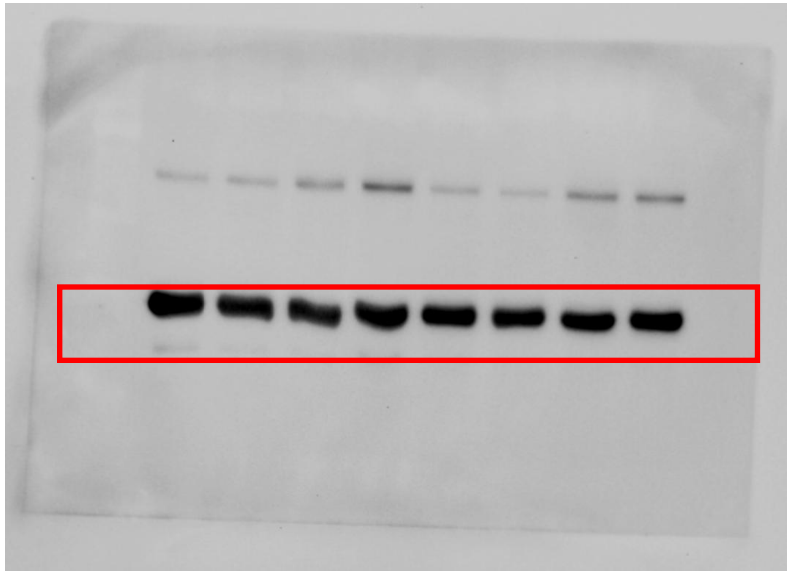

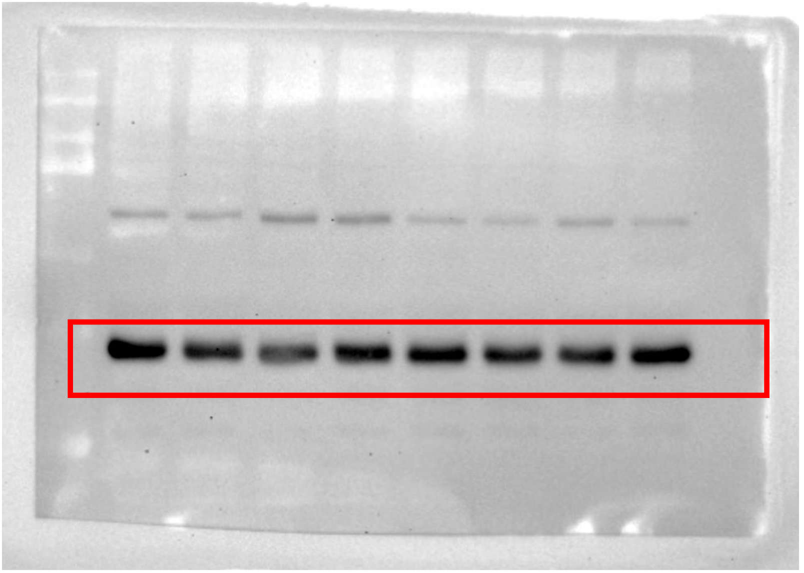

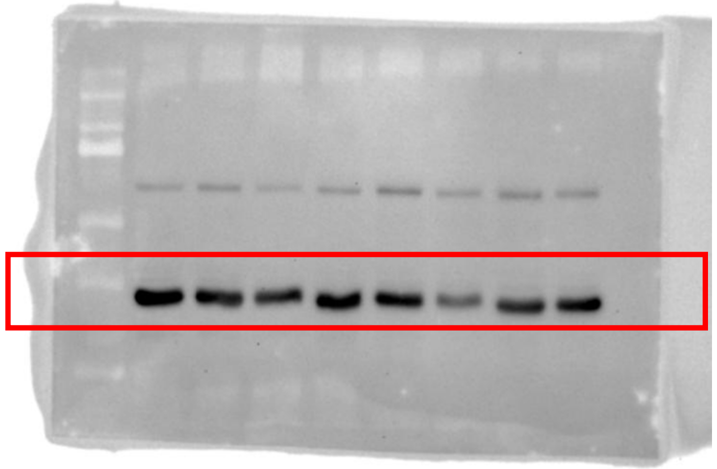

1-2 3-4 5-6

Beclin1

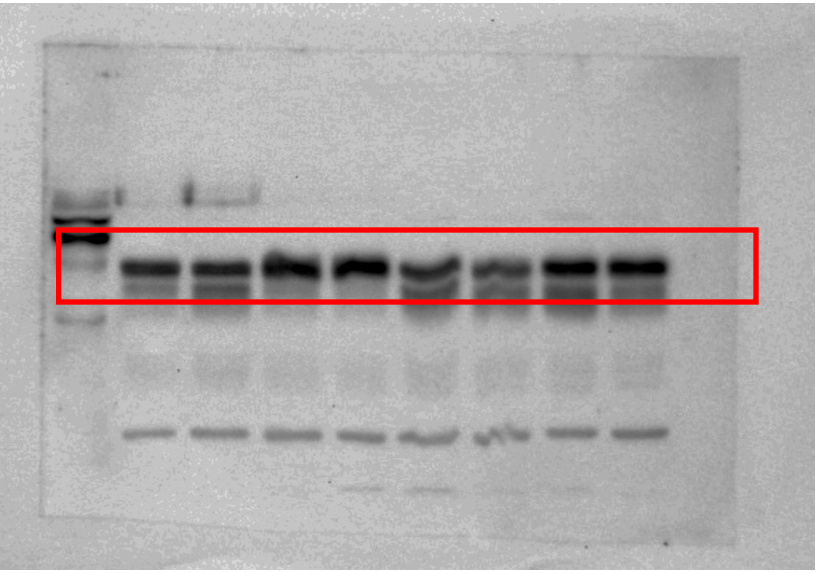

1-2

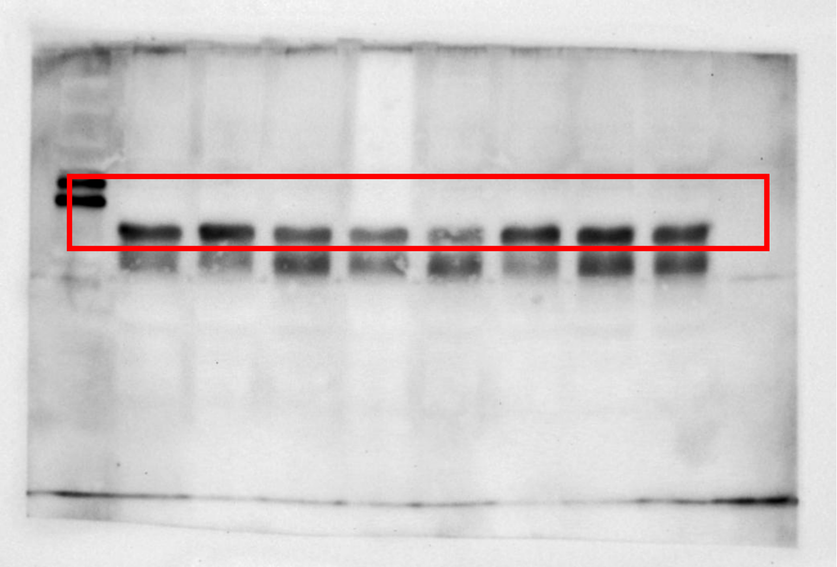

3-4

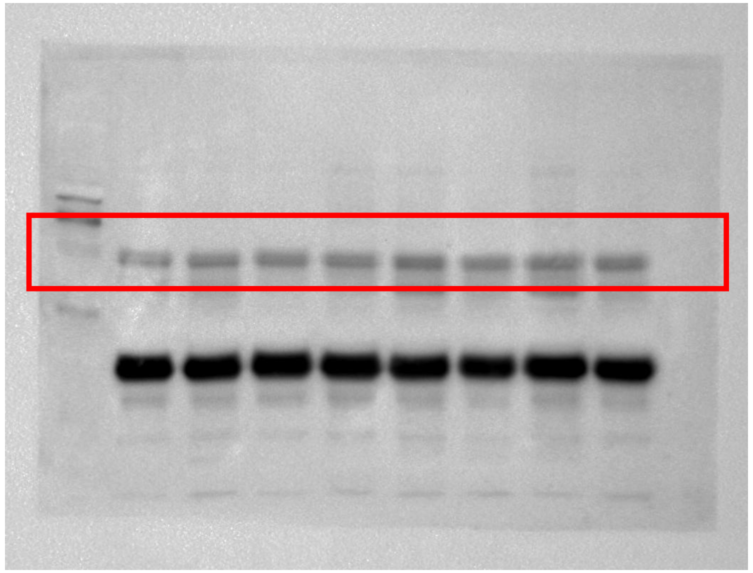

5-6

Beclin1(GAPDH)

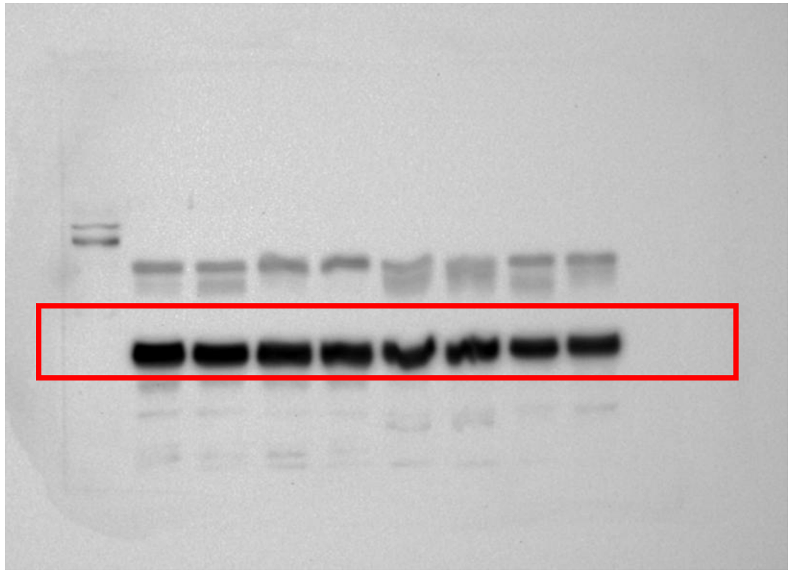

1-2

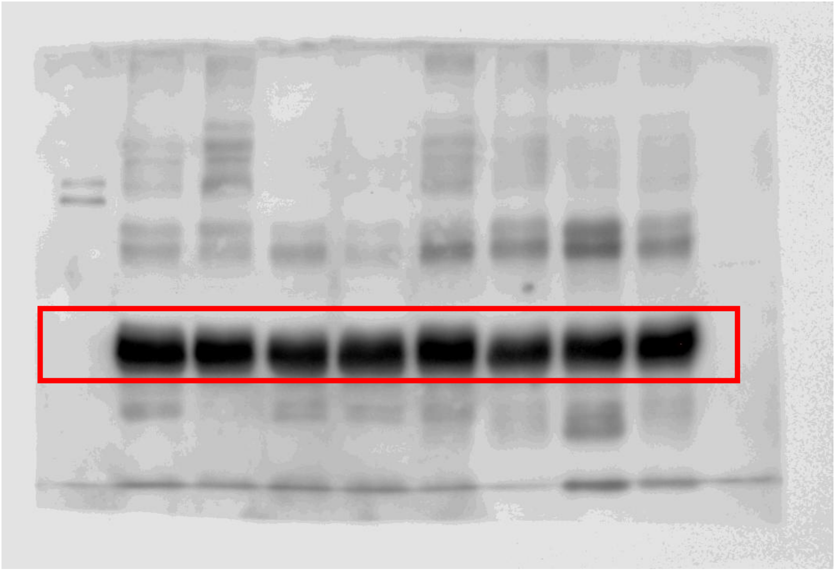

3-4

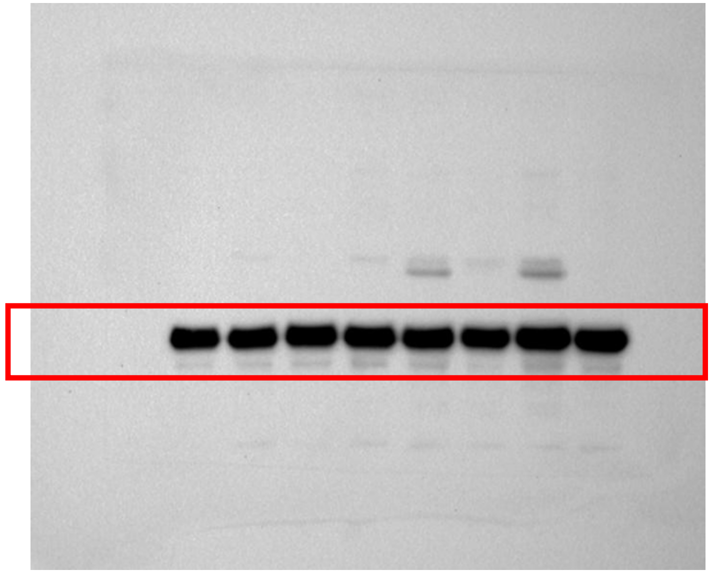

5-6

HIF1α

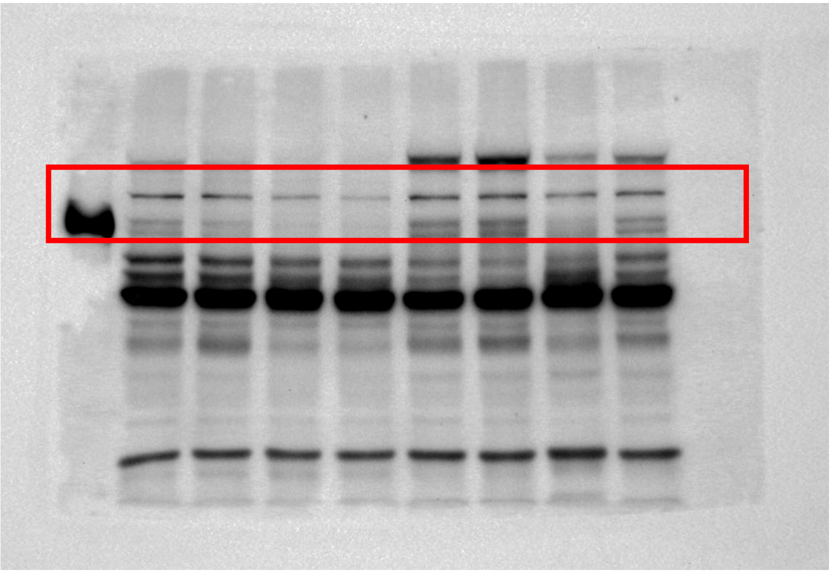

1-2

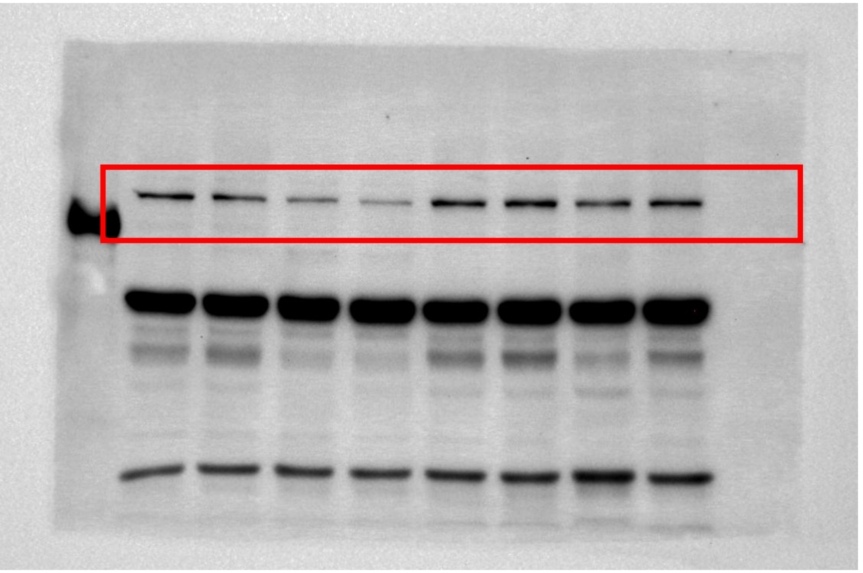

3-4

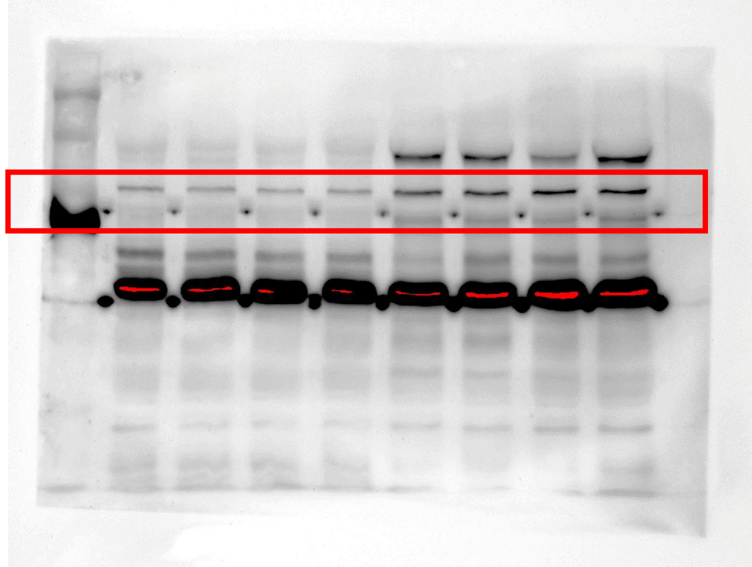

5-6

HIF1α(GAPDH)

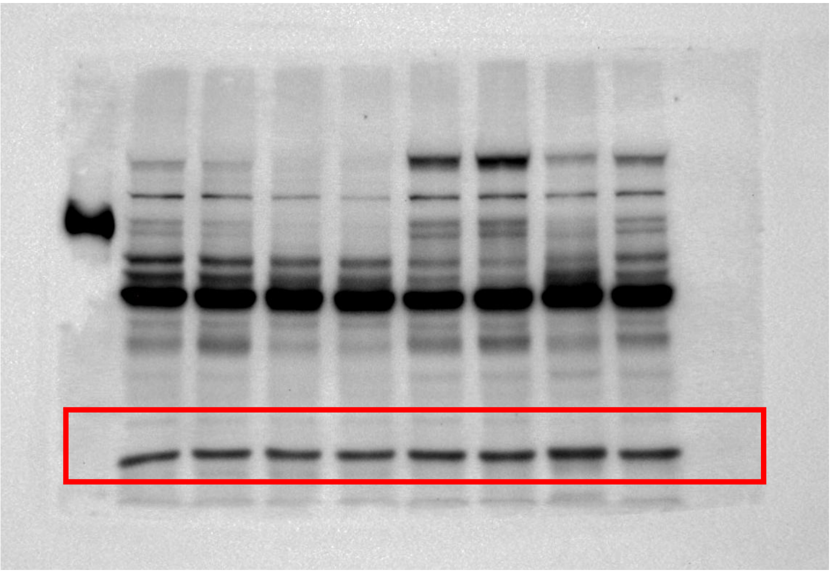

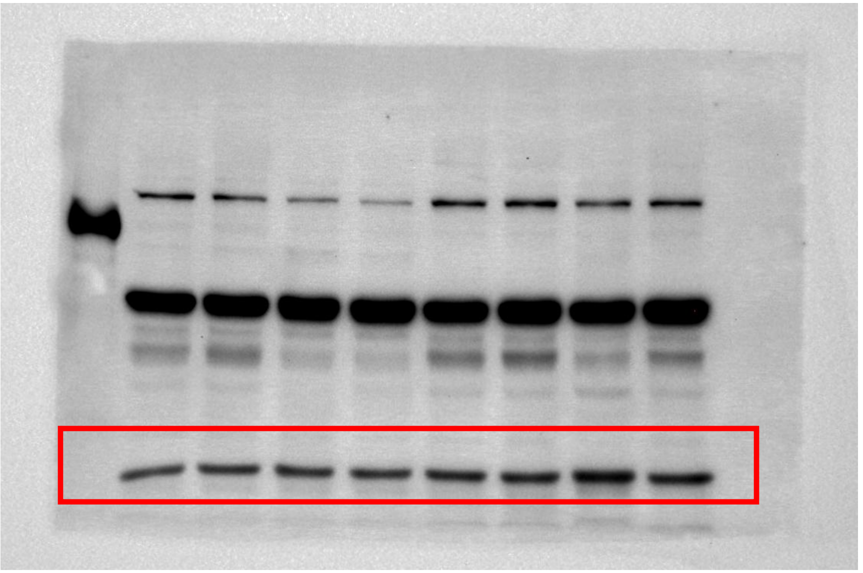

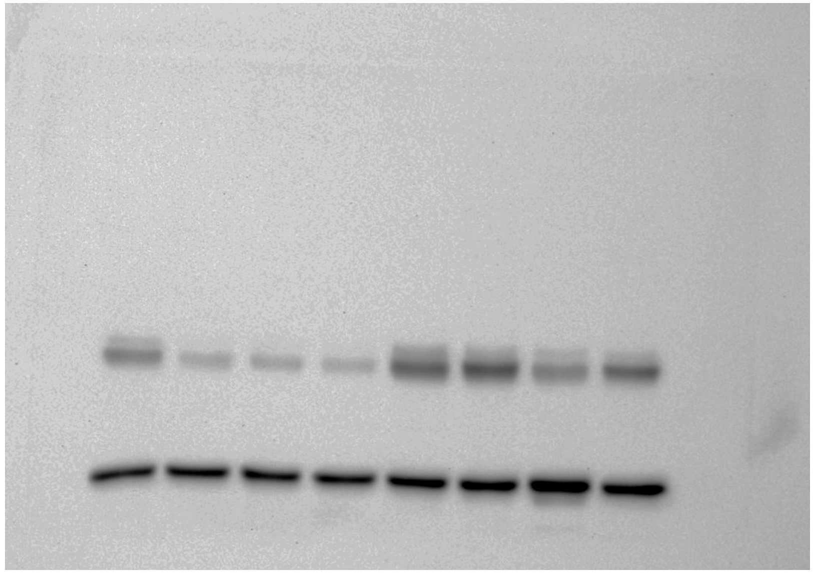

1-2 3-4 5-6

IL-1β

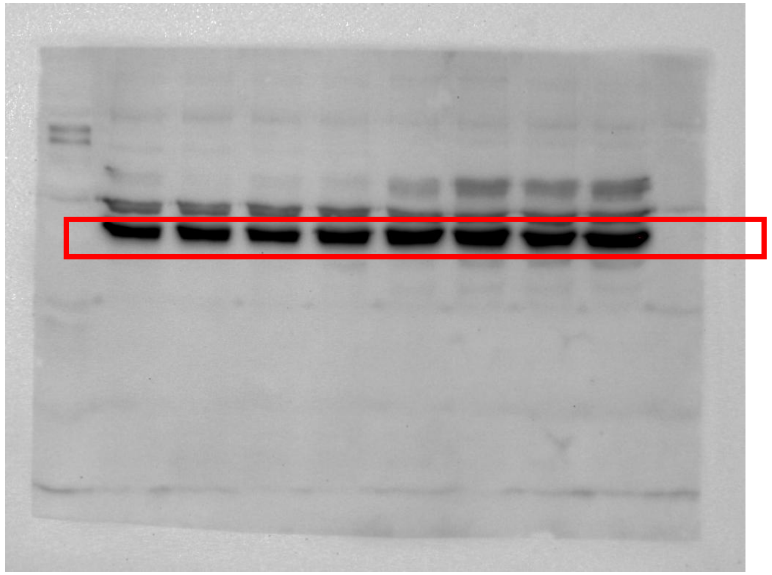

1-2

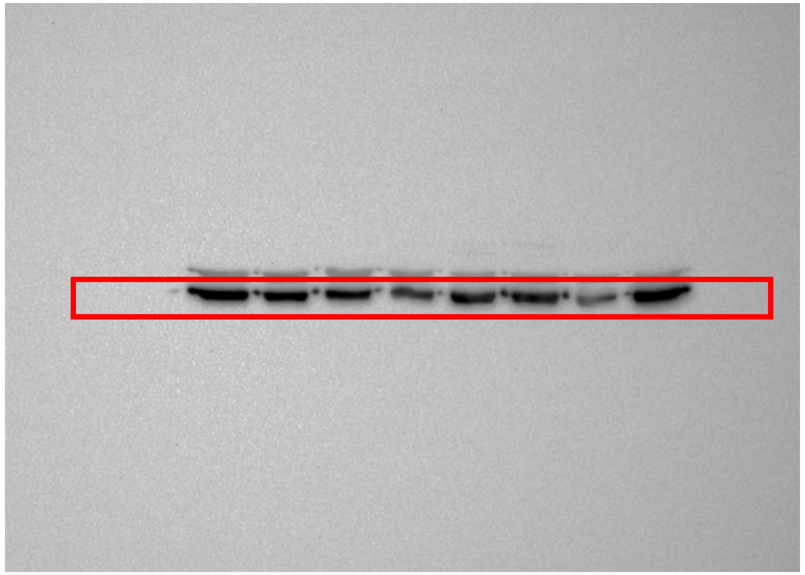

3-4

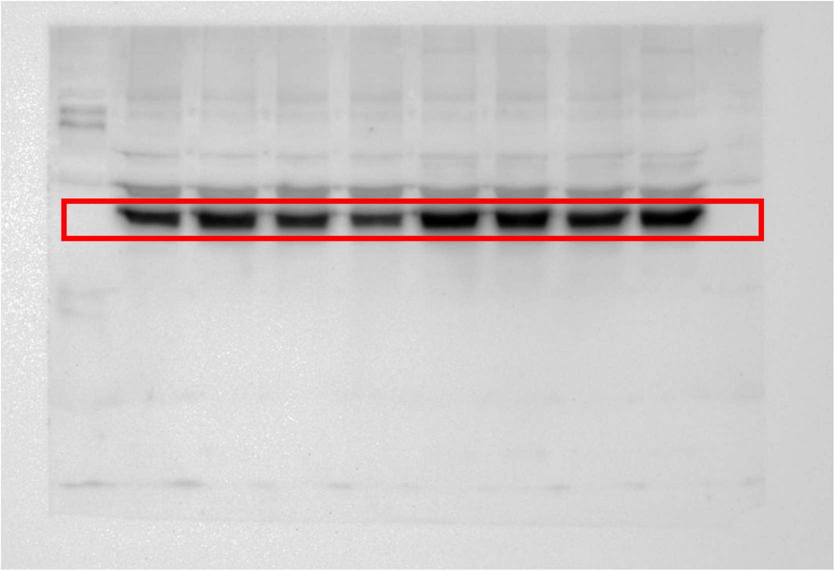

5-6

IL-1β (GAPDH)

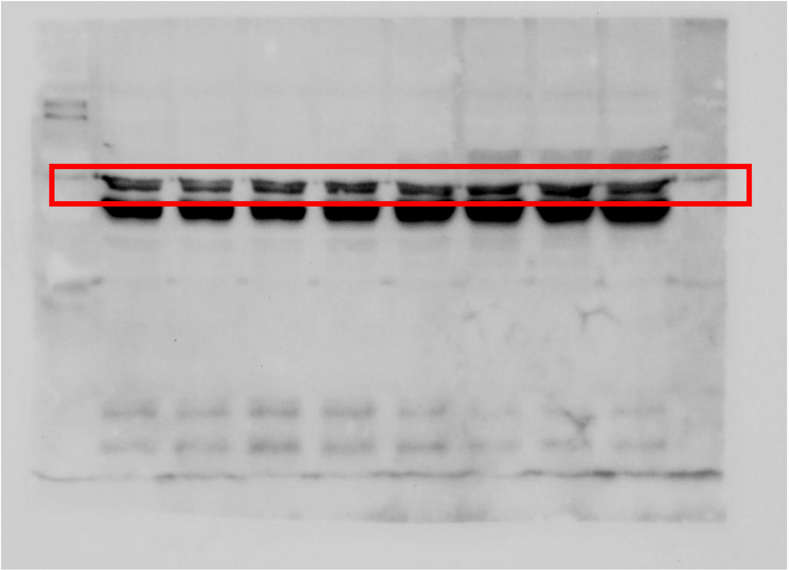

1-2

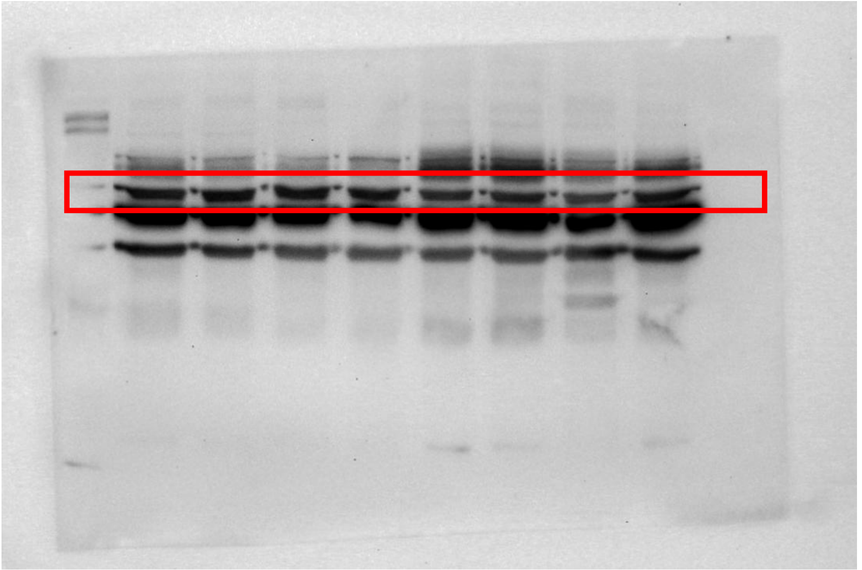

3-4

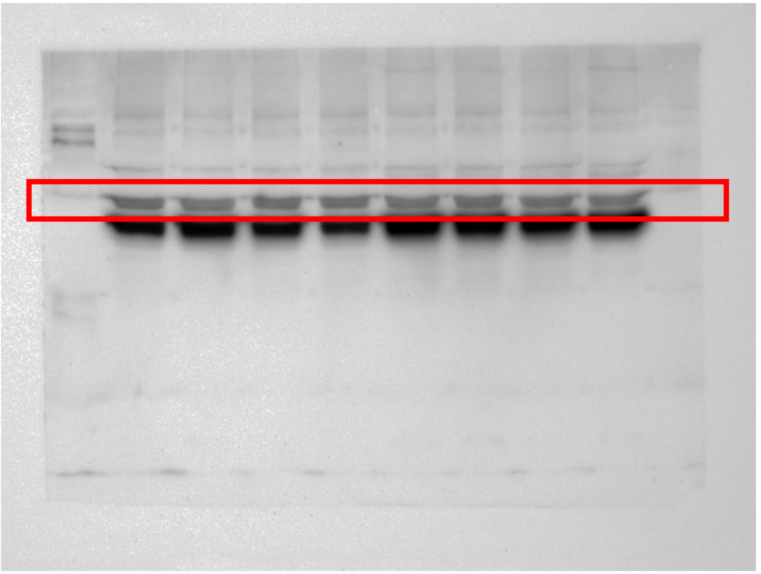

5-6

LC3B

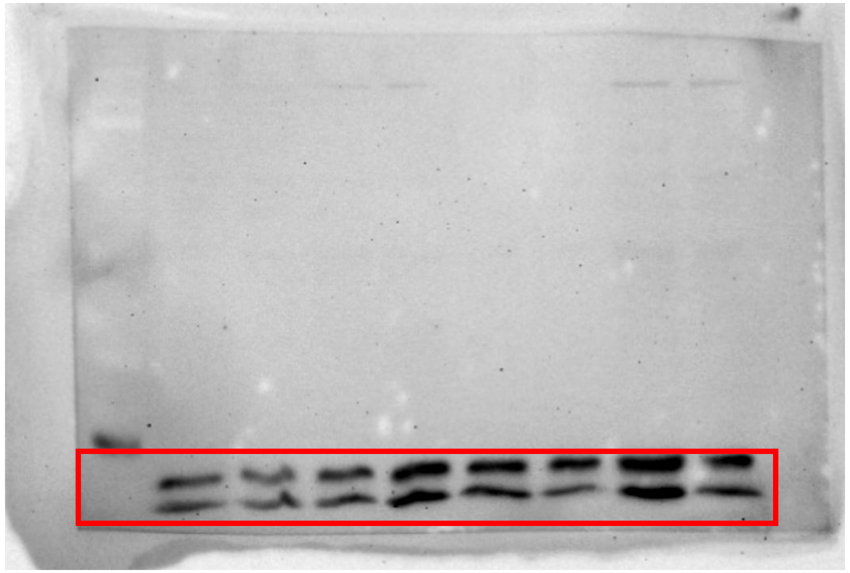

1-2

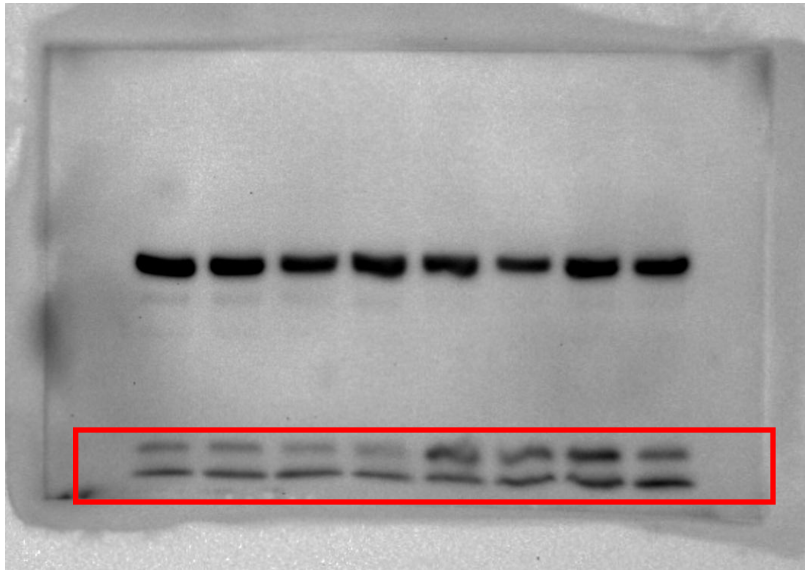

3-4

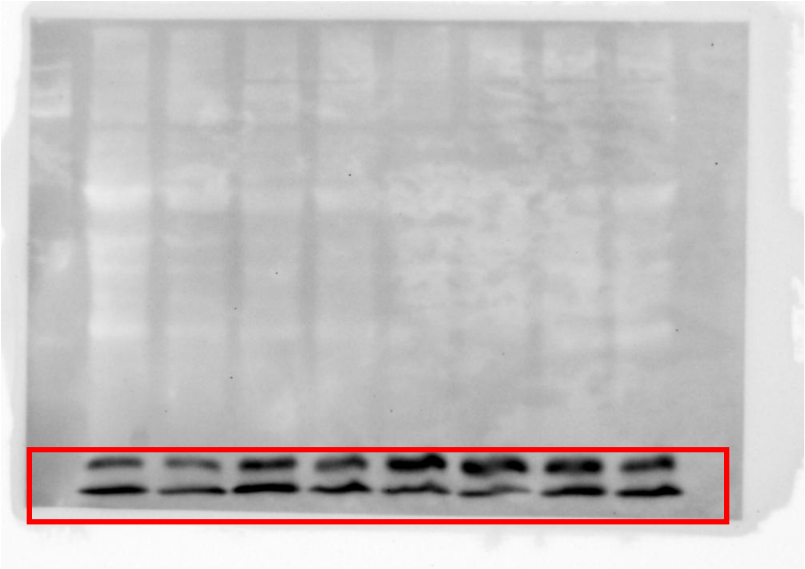

5-6

LC3B（GAPDH）

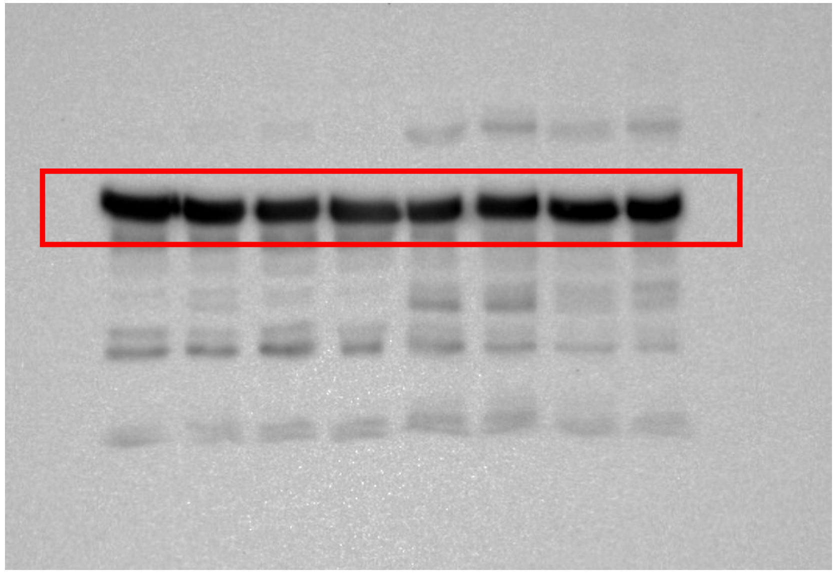

1-2

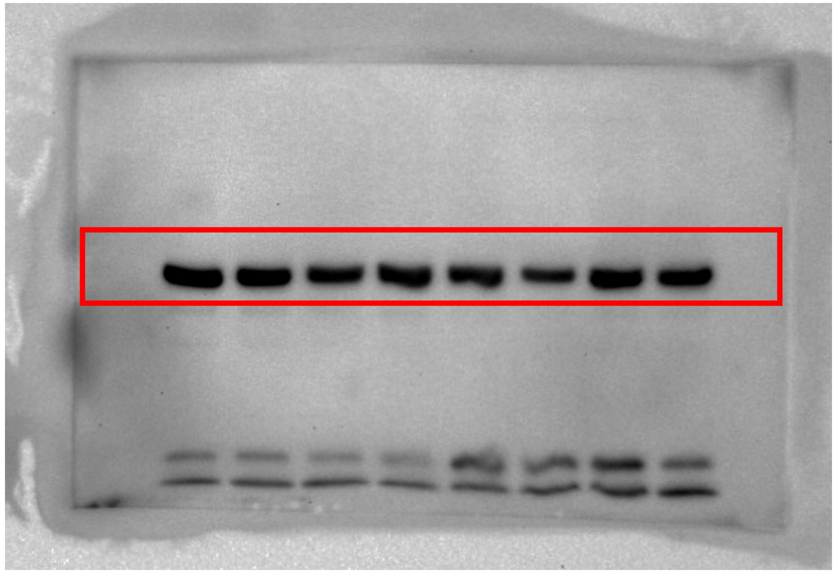

3-4

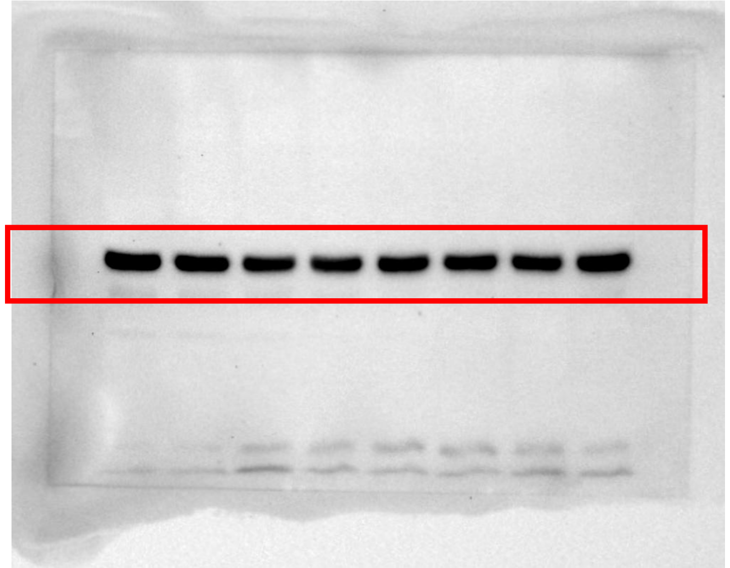

5-6

mTOR

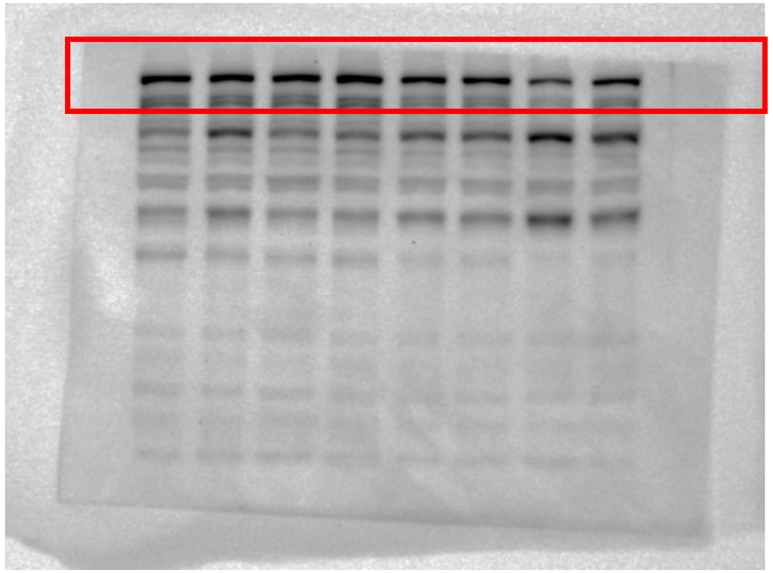

1-2

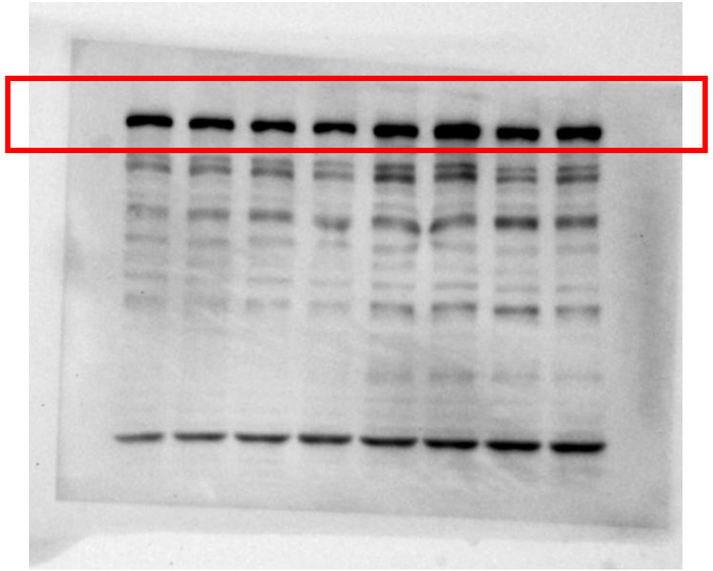

3-4

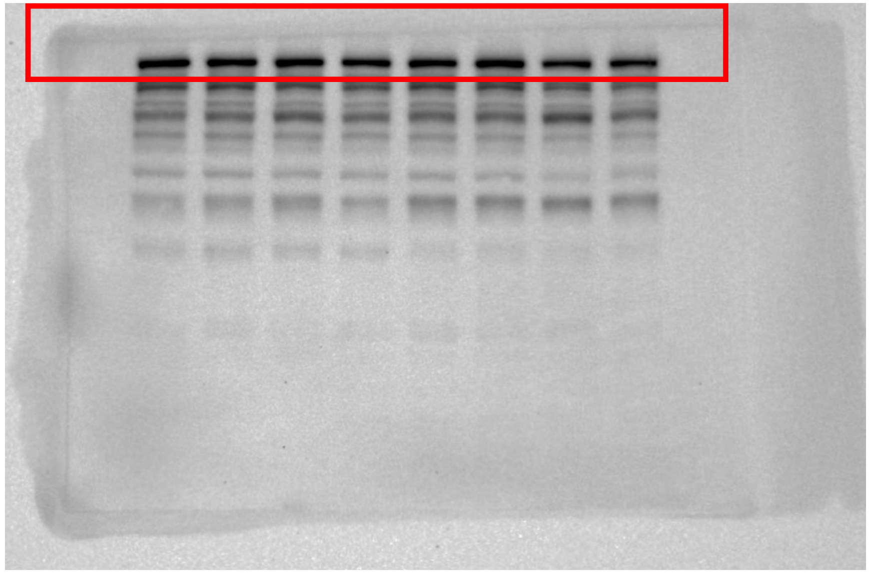

5-6

mTOR(GAPDH)

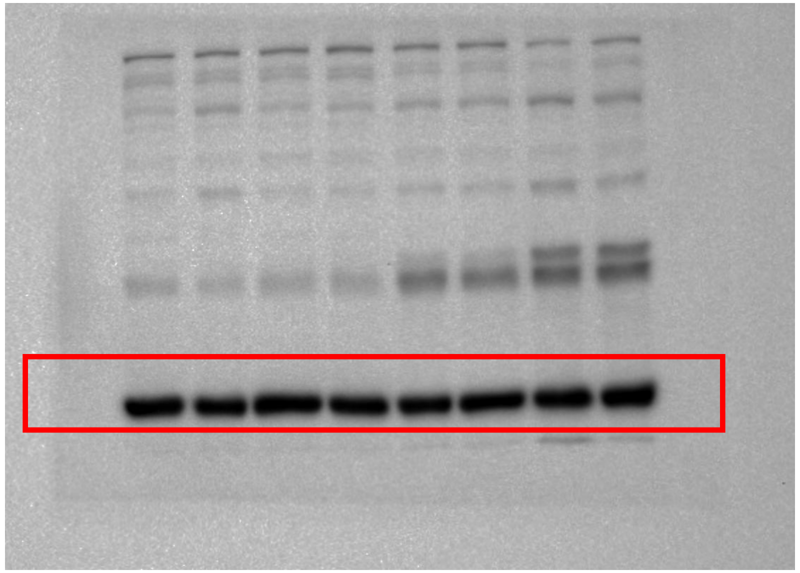

1-2

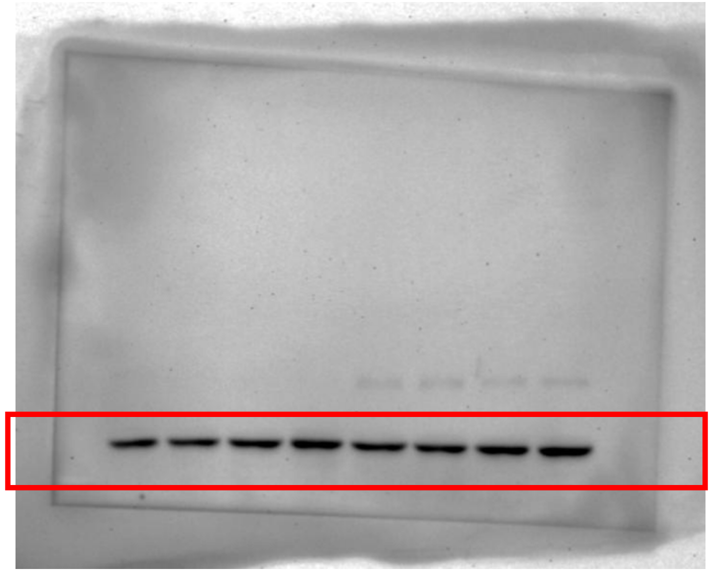

3-4

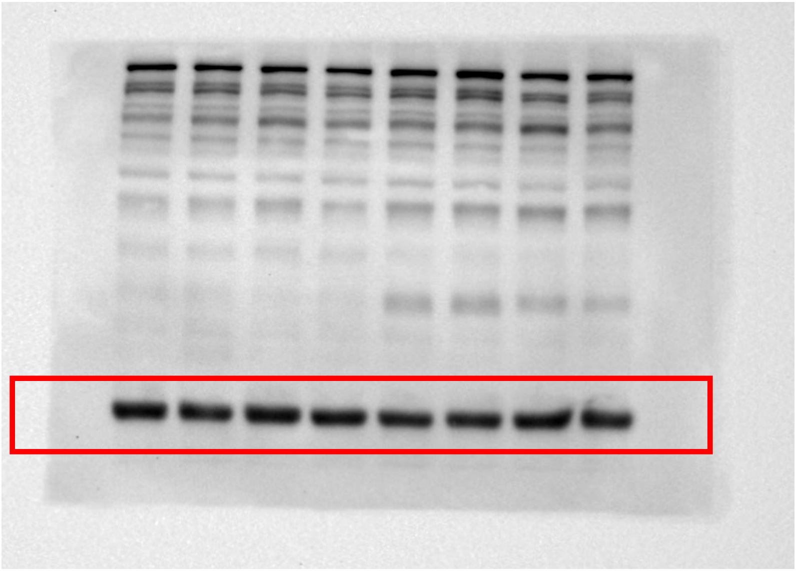

5-6

p62

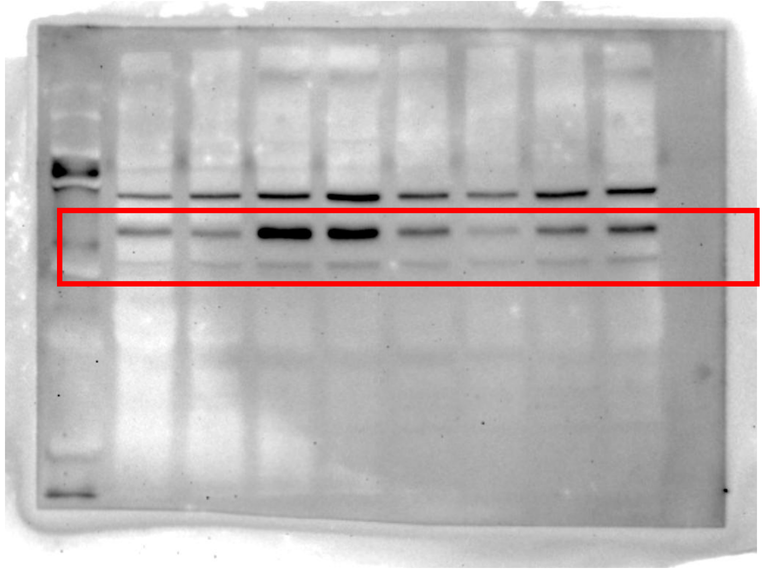

1-2

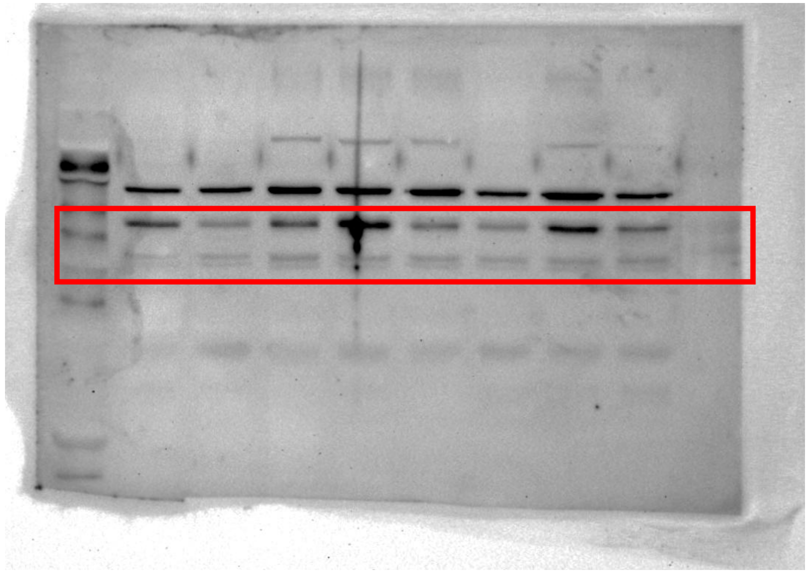

3-4

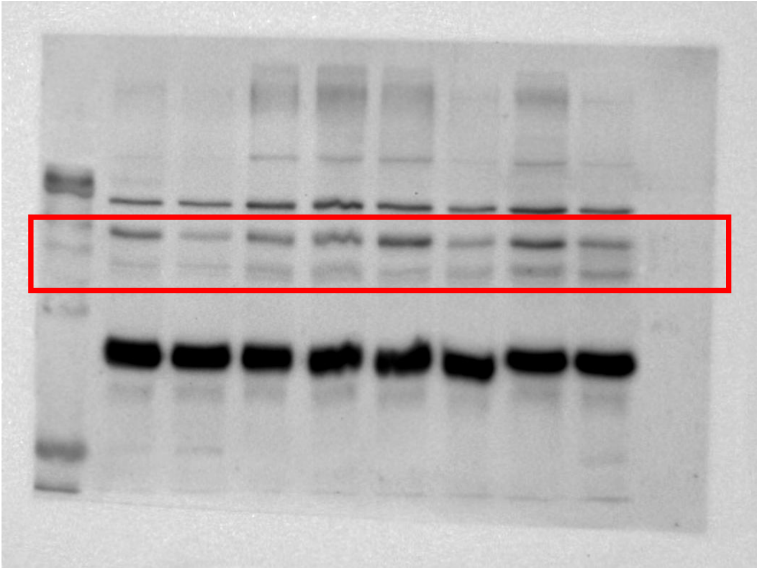

5-6

p62(GAPDH)

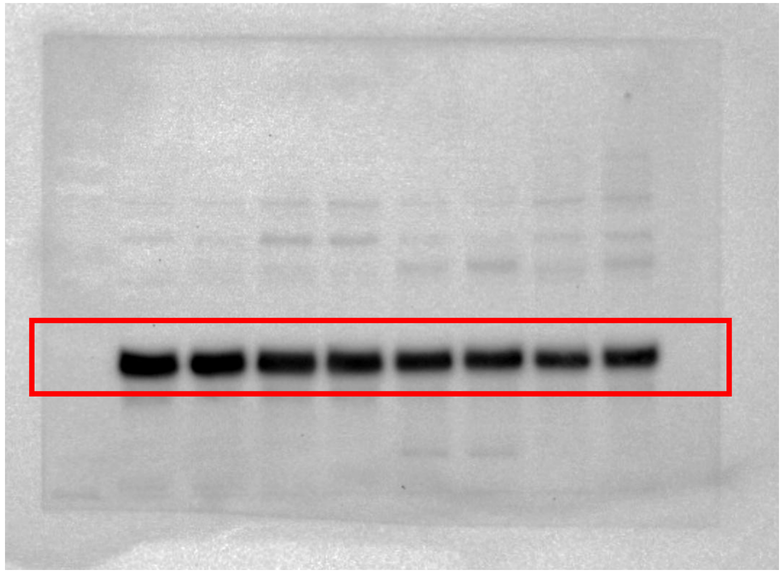

1-2

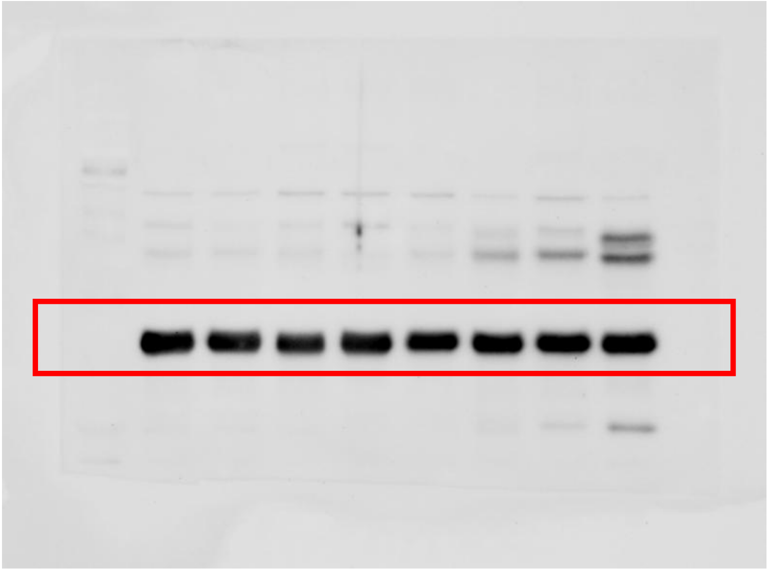

3-4

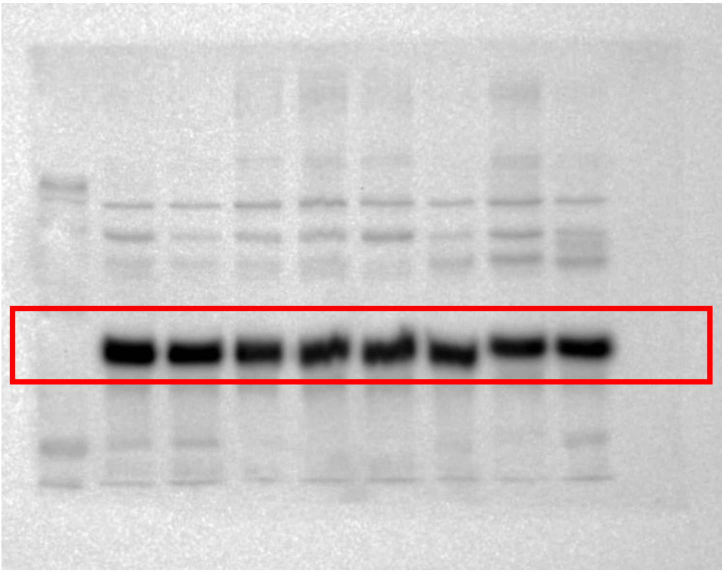

5-6

p70S6K

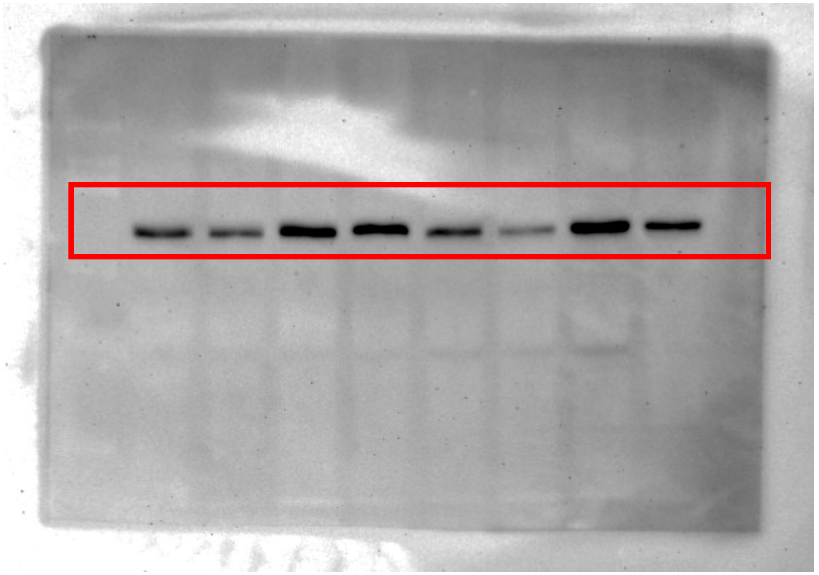

1-2

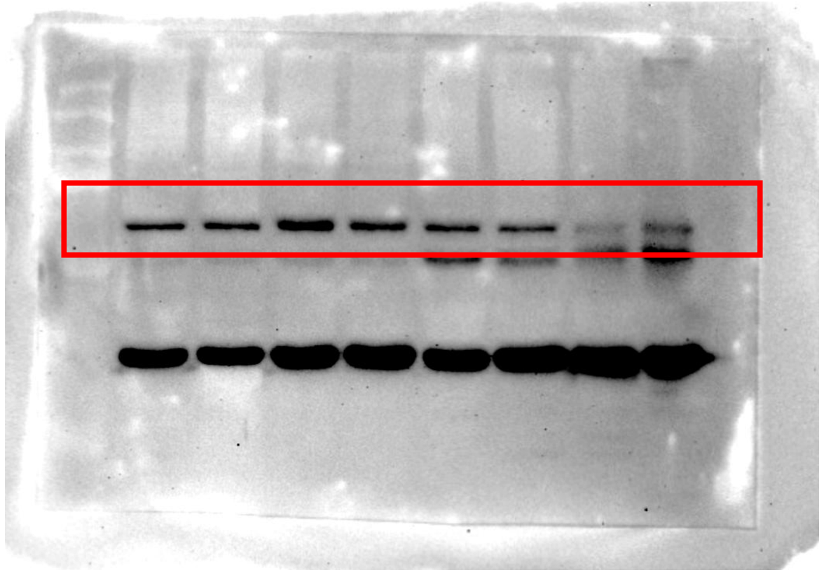

3-4

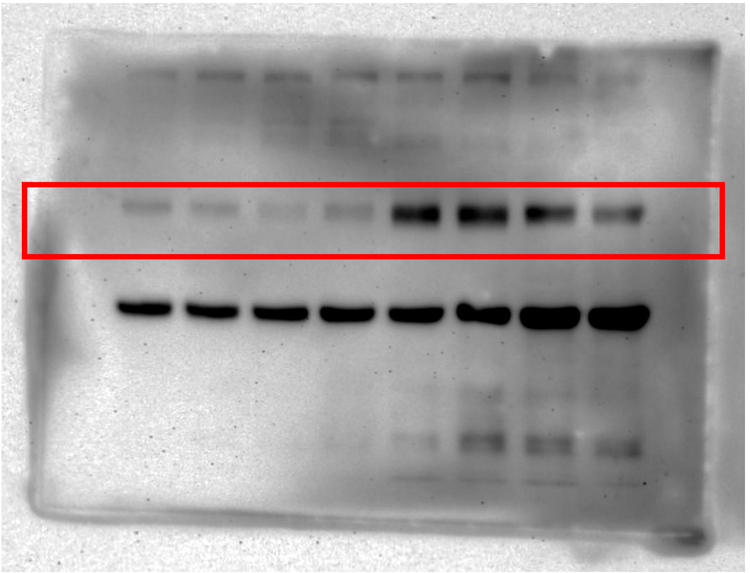

5-6

p70S6k(GAPDH)

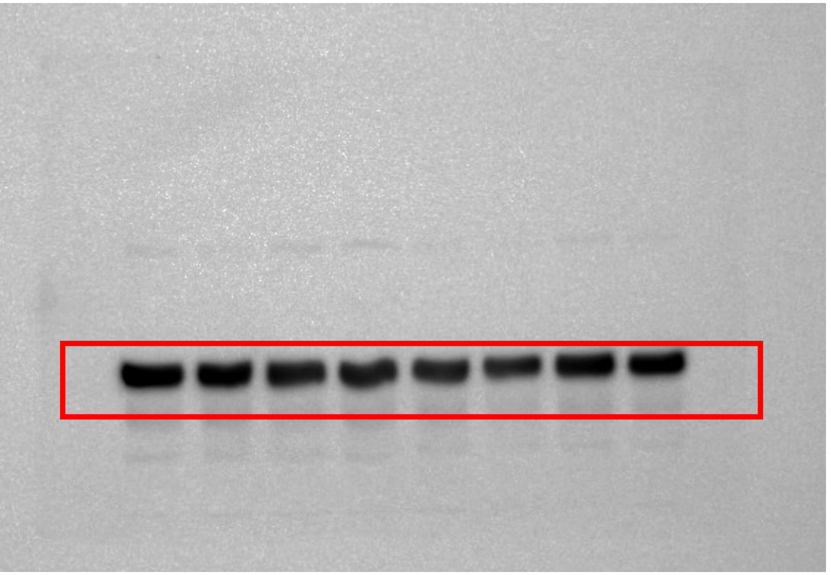

1-2

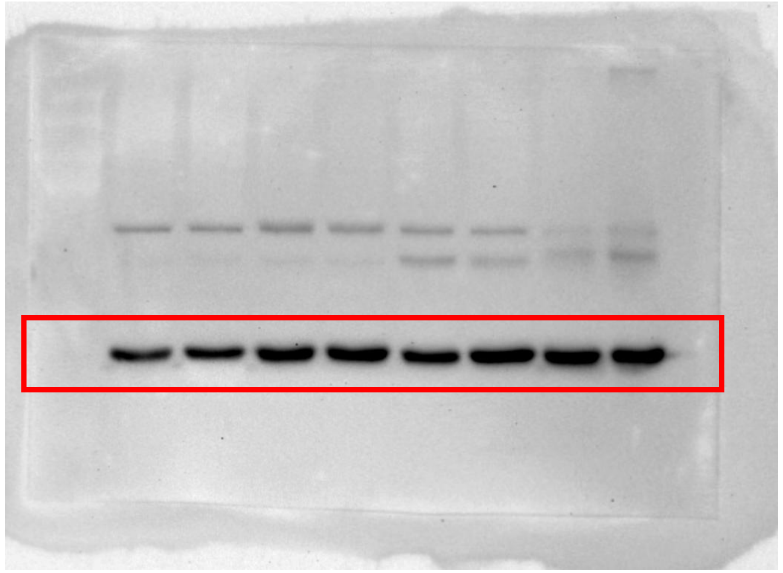

3-4

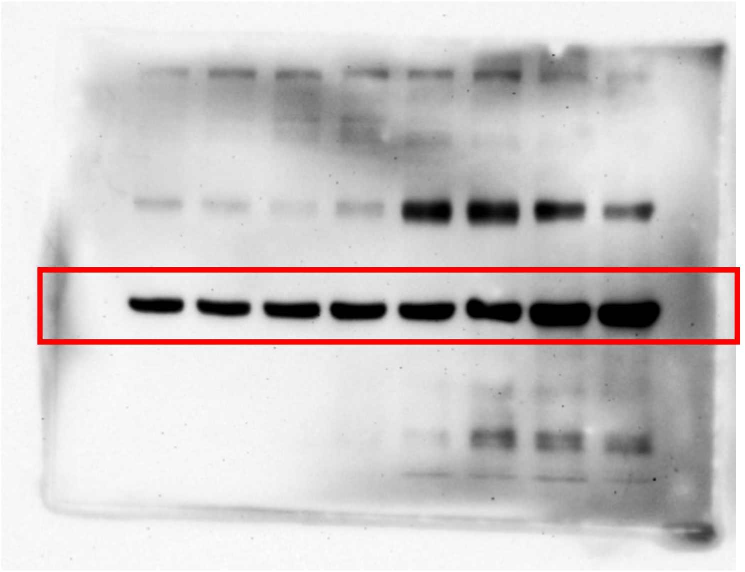

5-6

p-AMPK

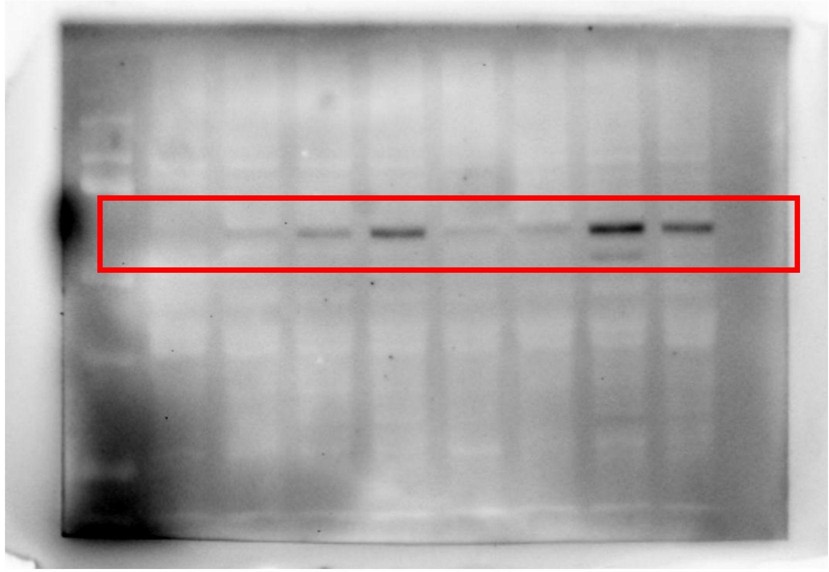

1-2

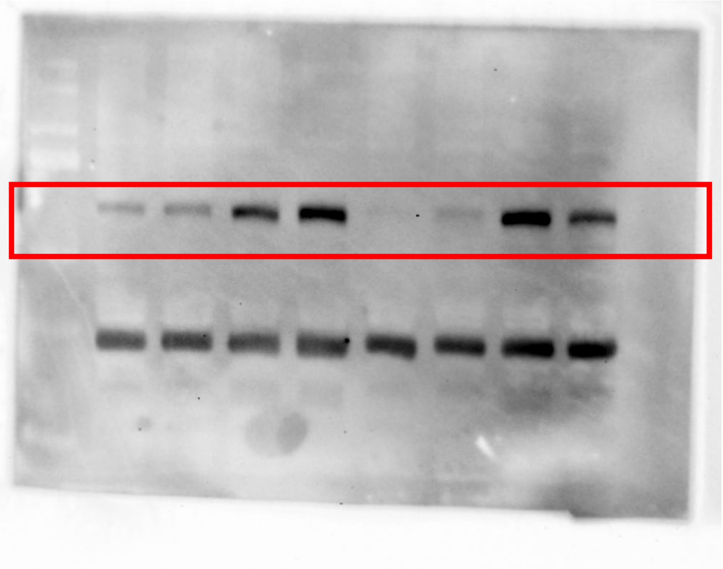

3-4

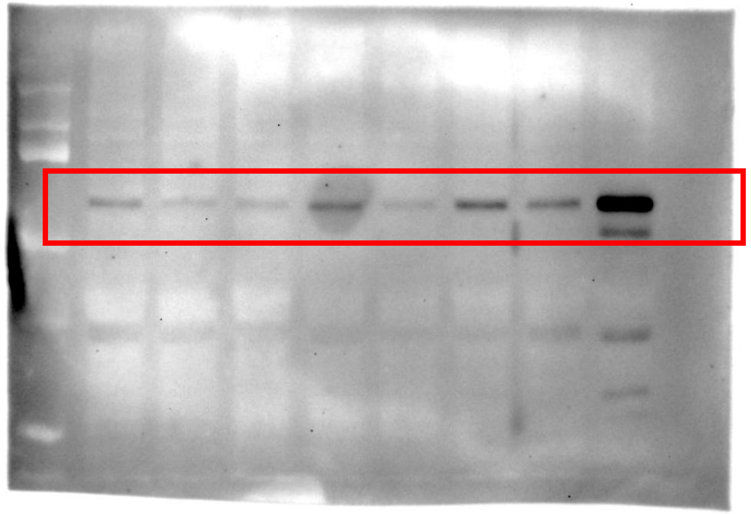

5-6

p-AMPK(GAPDH)

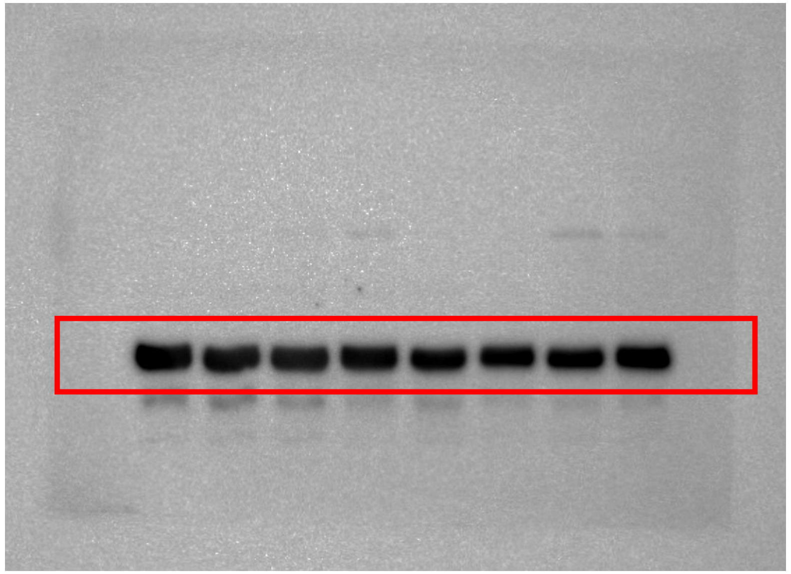

1-2

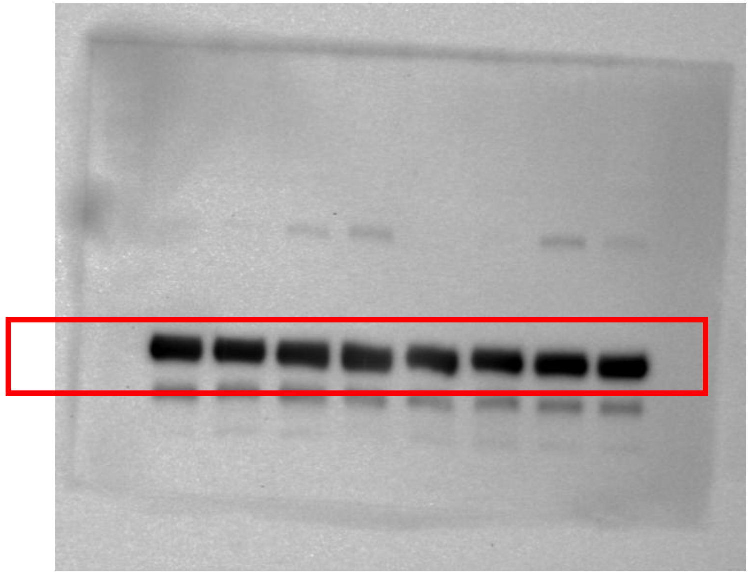

3-4

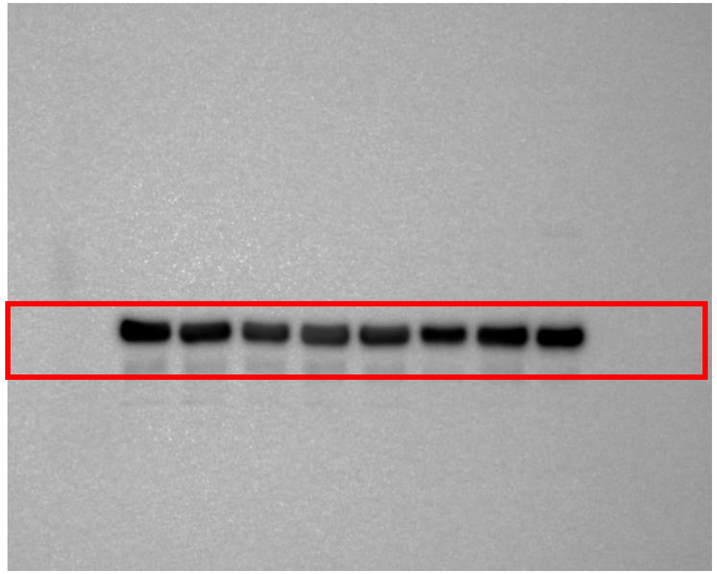

5-6

Raptor

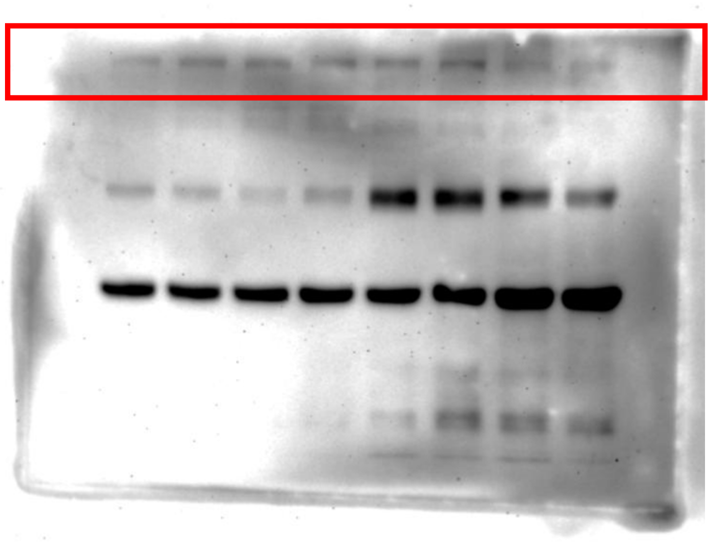

1-2

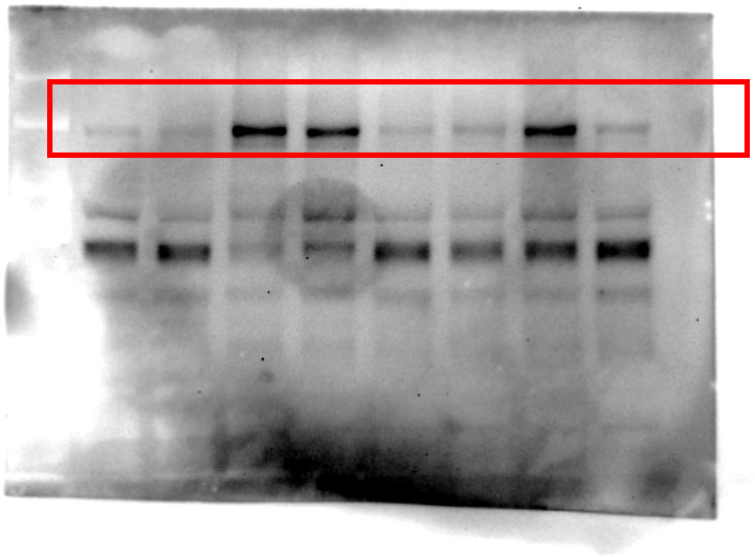

3-4

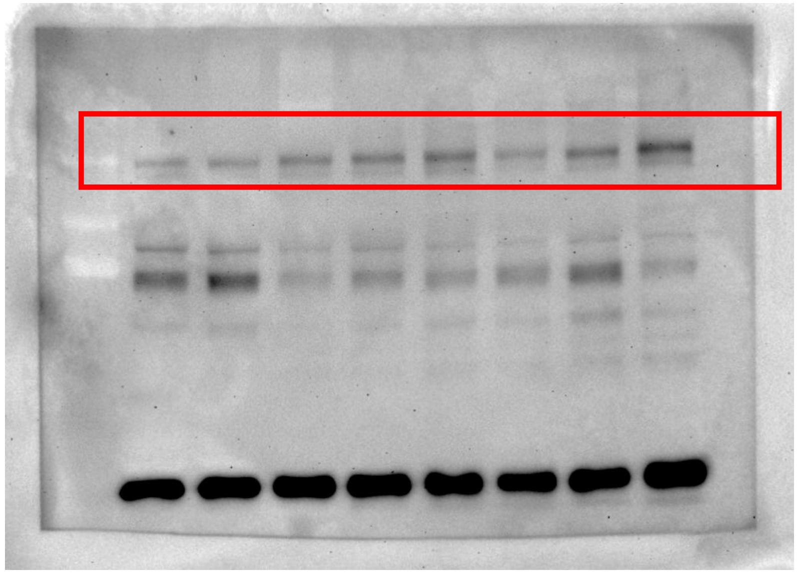

5-6

Raptor(GAPDH)

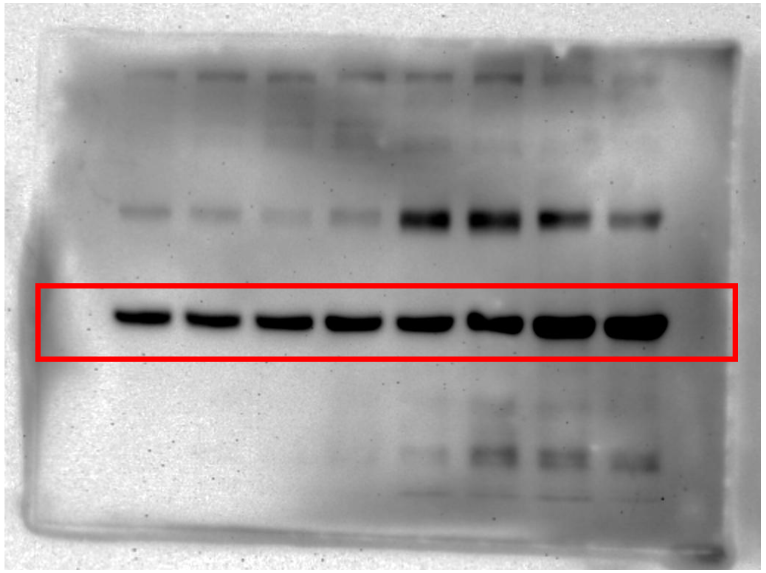

1-2

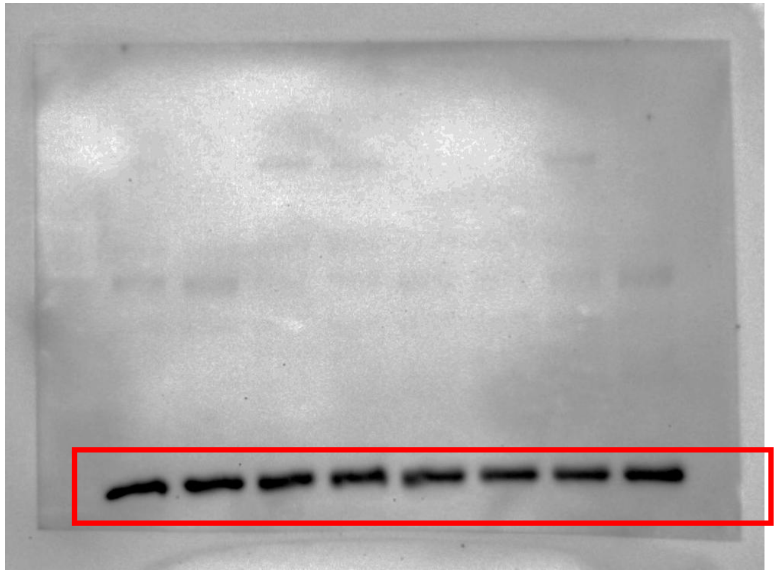

3-4

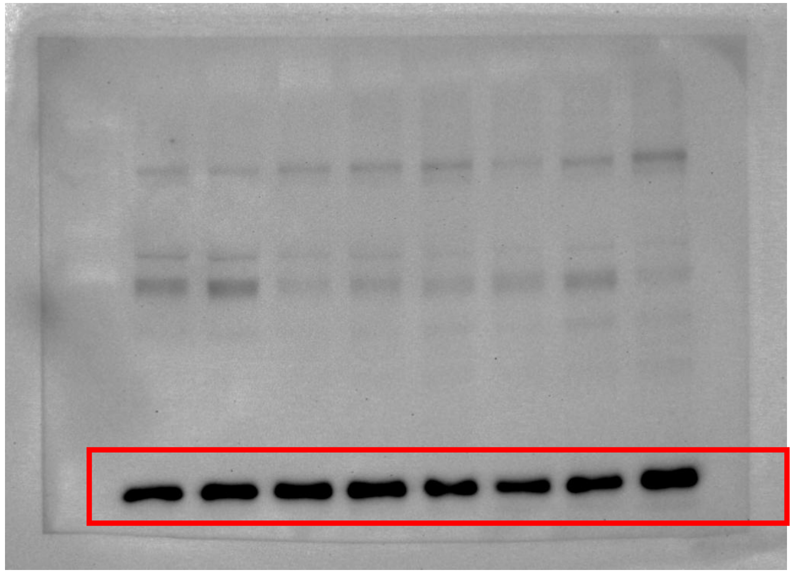

5-6

TFEB

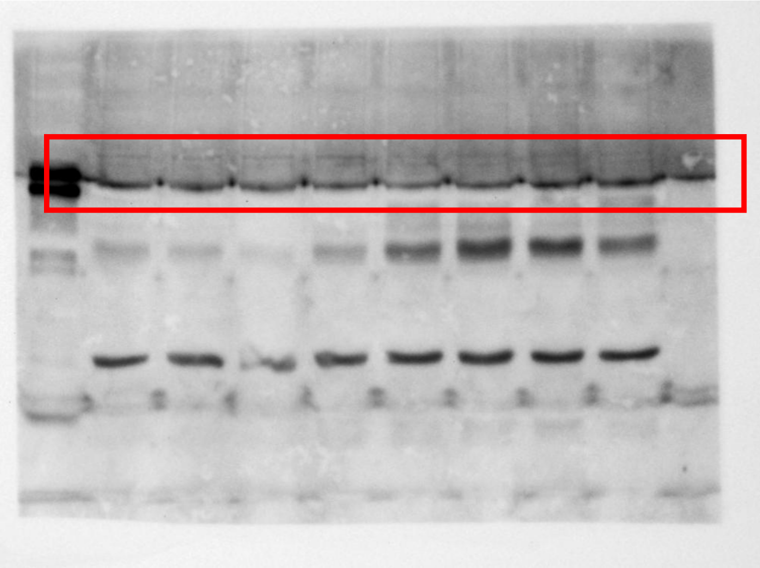

1-2

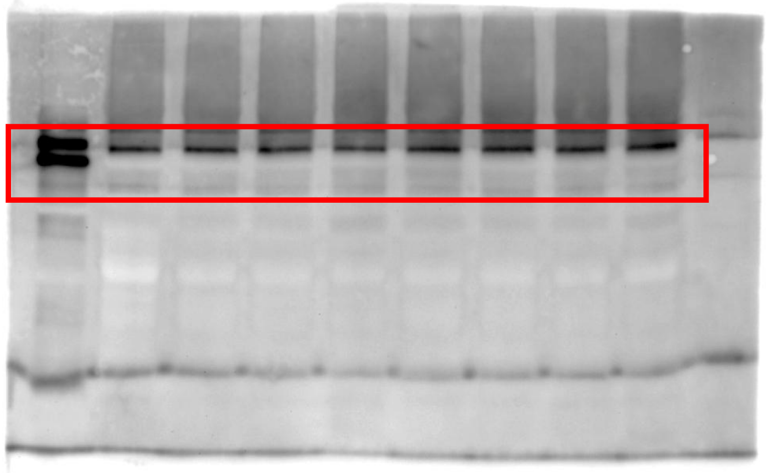

3-4

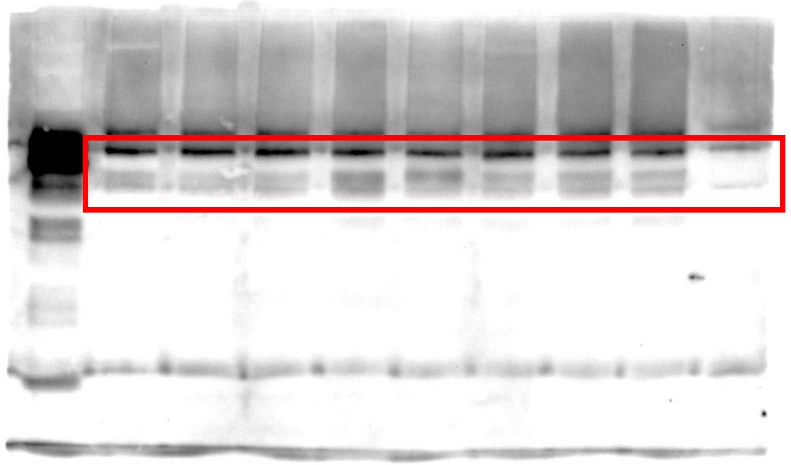

5-6

TFEB(GAPDH)

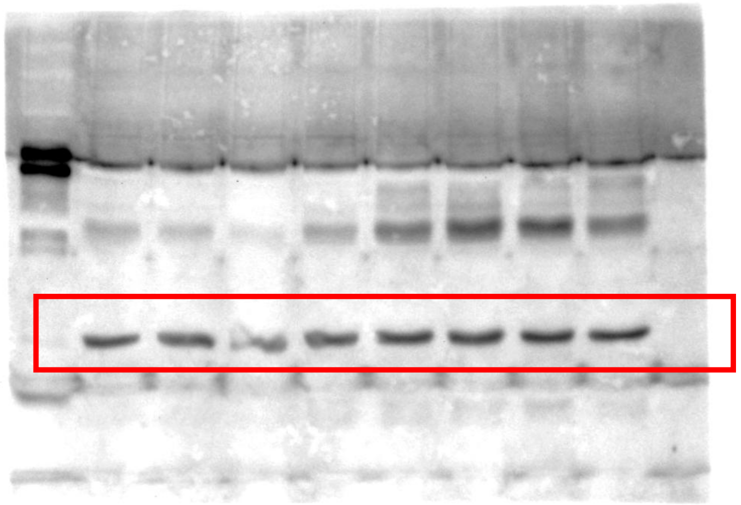

1-2

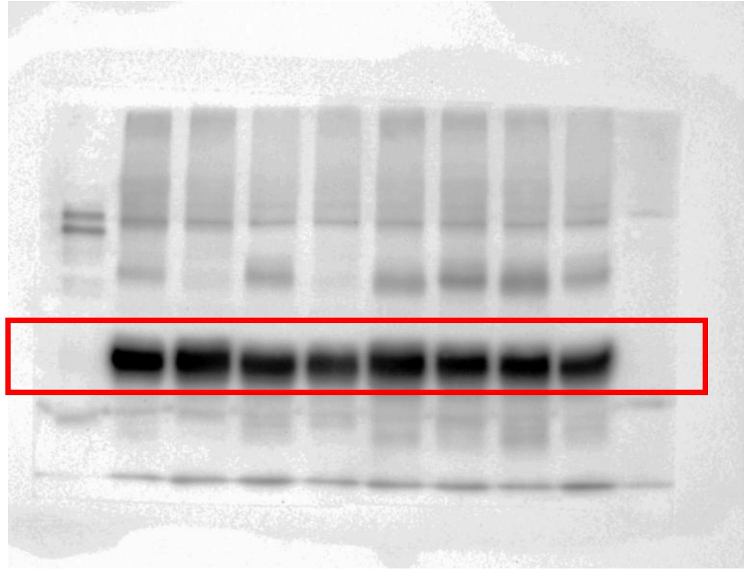

3-4

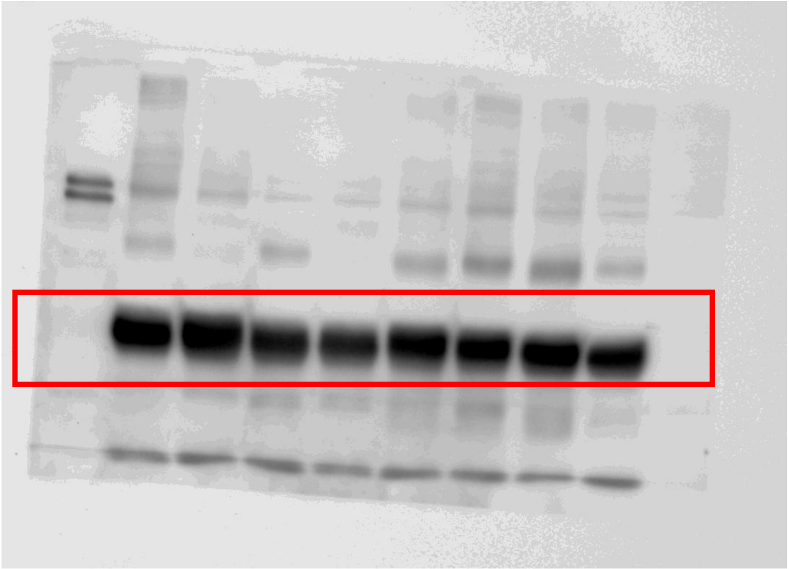

5-6
