# Supplementary material for: Resistance training alleviates muscle atrophy and muscle dysfunction by reducing inflammation and regulating compromised autophagy in aged skeletal muscle
Source: Front Immunol. 2025 Jun 3;16:1597222. doi: 10.3389/fimmu.2025.1597222 (PMC12170331; doi:10.3389/fimmu.2025.1597222)
Supplement: Supplementary file 2 [file Table1.docx]

Supplementary Material

**Tables**
Table 1：Sequences of forward and reverse primers used for qRT-PCR

| Gene | Primer forward | Primer reverse |
| --- | --- | --- |
| GAPDH | ACCCTTAAGAGGGATGCTGC | CCCAATACGGCCAAATCCGT |
| TNFα | GATCGGTCCCCAAAGGGATG | CCACTTGGTGGTTTGTGAGTG |
| NK-κB | CTCTGGCACAGAAGTTGGGT | TCCCGGAGTTCATCTCATAGT |
| IL-1β | TGCCACCTTTTGACAGTGATG | TGATGTGCTGCTGCGAGATT |
| IL-6 | GACAAAGCCAGAGTCCTTCAGA | TGTGACTCCAGCTTATCTCTTGG |
| IL-10 | GGTAGAAGTGATGCCCCAGG | ACACCTTGGTCTTGGAGCTTAT |

**Figure legends:
Figure 1

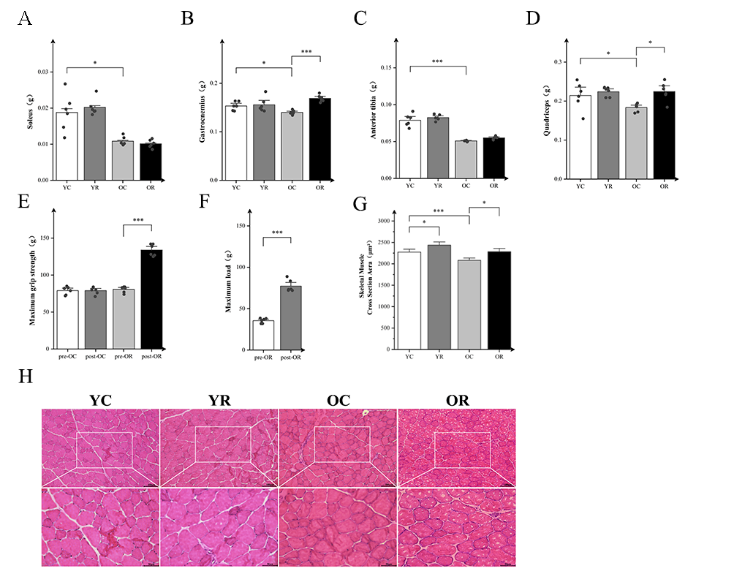

Figure 1** **Resistance Training increased wet weight, function and ameliorated CSA in aged skeletal muscle.** (A-D) Wet weights of the soleus (SOL), gastrocnemius (GAS), tibialis anterior (TA), and quadriceps (Quad). (E) Maximum grip strength. (F) Maximum load. (G) Cross-sectional area (CSA) of muscle fibers(μm²); (H) H&E staining.

**Figure 2**

**
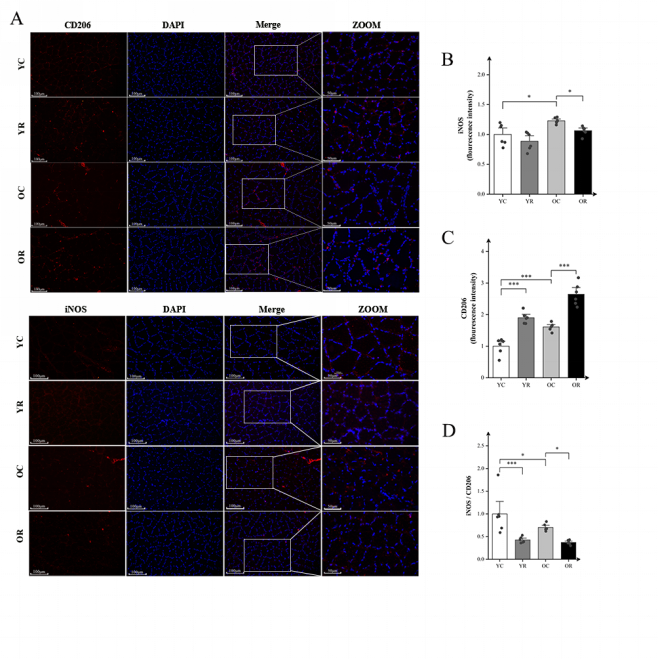
****Figure 2. Resistance training promoted polarization macrophages toward the anti-inflammatory phenotype in aged skeletal muscle.** (A) Representative fluorescence images of iNOS. (B) Fluorescence intensity expression of iNOS. (C) Representative fluorescence images of CD206. (D) Fluorescence intensity expression of CD206. All data are presented as means ± SEM. Six random regions were quantified for each sample, n=6. *p<0.05, **p<0.01, ***p<0.001.

**Figure 3****
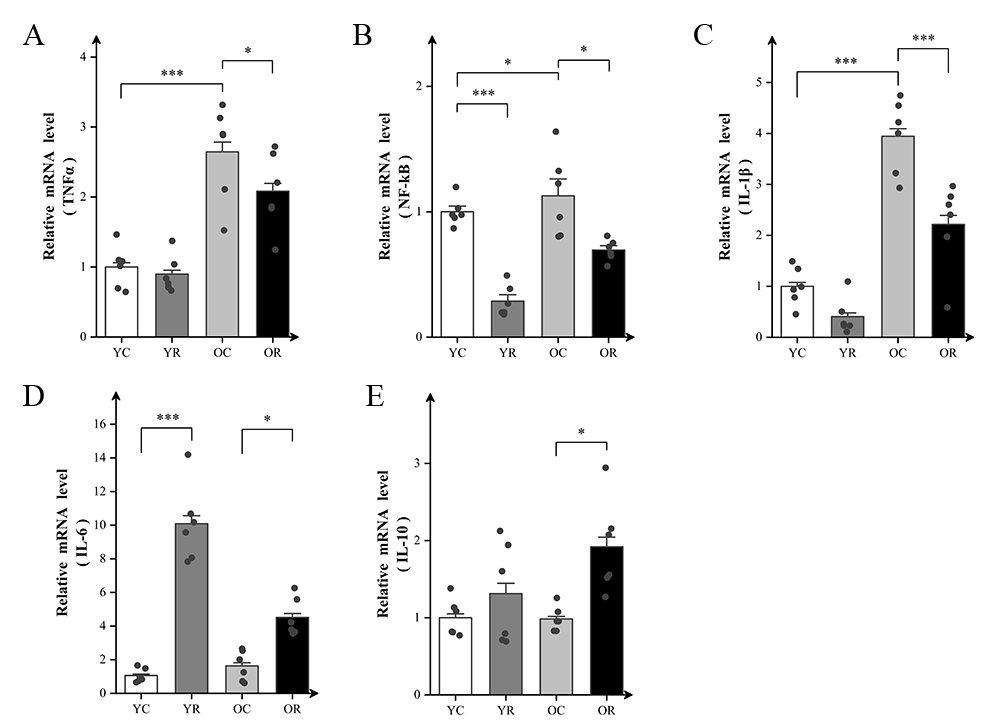

Figure 3 Resistance training attenuated mRNA levels of inflammatory cytokines in aging skeletal muscle.** (A-E) The mRNA expression of TNFα, NF-kB, IL-1β, IL-6 and IL-10. Data are expressed as fold change from rest and error bars represent SEM, n=6. *p<0.05, ***p<0.001.

**Figure 4****
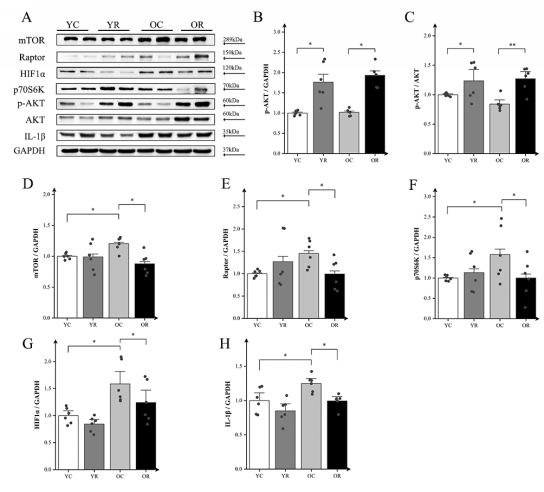

Figure 4. Resistance training regulated mTORC1-HIF-1α pathway and alleviated chronic inflammation.** (A) Representative Western blot images of mTOR, Raptor, HIF-1α, P70S6K, p-AKT, AKT, IL-1β, and the internal control GAPDH in the quadriceps muscle. (B-H) Protein expression levels of mTOR, Raptor, HIF-1α, P70S6K, p-AKT, AKT, and IL-1β. All data are presented as means ± SEM. Each group comprised n = 6; *p < 0.05, **p < 0.01, ***p < 0.001.

**Figure 5****
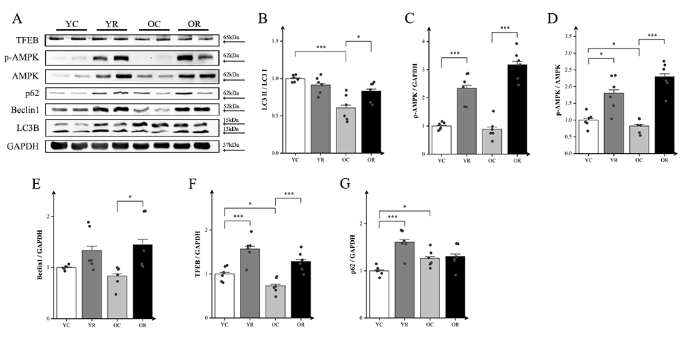

Figure 5. Resistance training activated autophagy and altered mTORC1-AMPK pathway.** (A) Representative Western blot images of TFEB, p-AMPK, AMPK, p62, Beclin1, LC3B, and the internal control GAPDH in the quadriceps muscle. (B-G) Protein expression levels of TFEB, p-AMPK, AMPK, p62, Beclin1, and LC3B. All data are presented as means ± SEM, n = 6. *p < 0.05, **p < 0.01, ***p < 0.001.
